# Histamine regulates the activity and the expression of the Na^+^/H^+^ exchanger (NHE)3 in human epithelial HK-2 cells

**DOI:** 10.1007/s00011-025-02095-4

**Published:** 2025-09-12

**Authors:** Chiara Gerbino, Federica Foglietta, Daniele Corsi, Patrizia Nardini, Luigi Cangemi, Elisa Benetti, Arianna Carolina Rosa

**Affiliations:** 1 Department of Scienza E Tecnologia del Farmaco, University of Turin, Via P. Giuria 9, 10125 Turin, Italy; 2 Iclas - GVM Care & Research, Via Mario Puchoz, 25, 16035 Rapallo, Italy; 3 Department of Clinical and Experimental Medicine, University of Florence, Cubo 2 - Viale Gaetano Pieraccini, 6, 50139 Florence, Italy

**Keywords:** Histamine, NHE3, Proximal tubule, ipH, BCECF-AM

## Abstract

**Objective and design:**

Investigate the potential role of histamine and its receptors on the functional expression of the sodium/hydrogen (Na^+^/H^+^) exchanger (NHE)3.

**Material:**

The human epithelial kidney (HK-2) cells were used as an in vitro model of the renal proximal tubule.

**Treatment:**

HK-2 cells were exposed to histamine 0–1000 nM alone or in combination with chlorphenamine (10 μM) and JNJ-7777120 (1 μM) for 0–48 h. MAPK involvement was determined using the selective inhibitors SB202190 (p38 MAPK), PD98059 (ERK1/2), and SP600125 (SAPK/JNK).

**Methods:**

Gene and protein expression were evaluated by qPCR and immunoblotting. The activity of NHE3 was measured by the BCECF-AM-based method.

**Results:**

Histamine (100 nM) induced a concentration-dependent NHE3 gene transcription with a peak 16 h after the treatment, followed by protein translation at 48 h after. A Consistent increase in NHE3 activity was observed at 48 h, but also at 60 min, when both p38 MAPK and ERK1/2 were phosphorylated. JNJ-7777120 blunted the activation and expression of NHE3. Chlorpheniramine was effective only on NHE3 activity.

**Conclusions:**

Histamine shows early (within 60 min) and late (48 h) effects on NHE3 expression. The histamine H_1_ and H_4_ receptors are shown to contribute to these effects differentially. The findings of this study extends the evidence for a direct contribution of histamine on the renal reabsorptive machinery.

**Supplementary Information:**

The online version contains supplementary material available at 10.1007/s00011-025-02095-4.

## Introduction

The Na^+^/H^+^ exchanger 3 (NHE3) belongs to the NHE superfamily, which comprises 9 distinct isoforms based on the order of their molecular cloning. Each isoform serves a different function [1, 2]. Specifically, NHE3 is an antiporter expressed primarily on the apical membrane of the proximal tubule in the kidney and the small intestine of the gastrointestinal tract. NHE3 is responsible for the reabsorption of approximately 50% of the NaCl and 70% of the NaHCO_3_ from the glomerular filtrate and NH₄ secretion [3]. This process is known as the electroneutral exchange of intracellular H⁺ for extracellular Na⁺, and it is a critical component of maintaining fluid balance, regulating blood pressure, and ensuring the body’s acid–base equilibrium. In addition to its role in urine pH modulation, NHE3 has been associated with the development of albuminuria, a condition characterised by the presence of albumin in the urine, due to its interaction with megalin, the protein responsible for albumin reabsorption in the proximal tubule [4].

Accumulating evidence supports the hypothesis that NHE3 plays a role in tubuloglomerular feedback. In this mechanism, the conveyance of NaCl at the macula densa leads to the constriction or dilation of the glomerular afferent arteriole in response to high or low NaCl concentrations, respectively, thus altering the glomerular filtration rate (GFR). NHE3 activity has been demonstrated to affect the concentrations of NaCl in the luminal fluid. An increased NHE3 activity has been shown to cause a reduction in the NaCl concentration, which, in turn, evokes afferent arteriole dilation and, ultimately, increases the GFR. NHE3^−/−^ total body mice, which exhibit increased intestinal luminal water content and diarrhea, demonstrate a reduced water flux across the proximal tubule and a decreased GFR [5–7]. The study by Li, et al. [8] shows that proximal tubule–specific deletion of NHE3 reduced basal blood pressure, urine osmolality, and GFR, as well as increased the pressure-natriuresis response, fluid intake, urinary flow rate, urinary sodium/creatinine, and urine pH. However, Poulsen, et al. [9] obtained different results, with NHE3^loxloxPax8^ mice displaying a normal GFR. Furthermore, Onishi, et al. [10] demonstrated that knocking down the tubular NHE3 in type 1 diabetic Akita mice did not affect hyperglycemia or diabetes-induced GFR, but had a significant effect on diabetes-independent GFR and prevented diabetes-associated albuminuria. NHE3 expression has been positively correlated to diabetic nephropathy since 1986 [11], and its role is confirmed by clinical evidence of increased tubular reabsorption in Type 1 [12] and Type 2 [13] diabetic patients. It has been demonstrated that insulin [14], glucose [15], and angiotensin II [1, 3, 16, 17] induce NHE3 overactivity/expression. As evidenced by numerous studies, anti-diabetic pharmaceuticals, including gliptins [18], incretin mimetics [19], and glifozines [20], as well as renin-angiotensin-system blockers [21, 22], also approved for diabetic nephropathy, cause a reduction in NHE3 expression and inhibit its activity. In particular, cardio-renal protection of gliflozins is exerted behind their anti-glycemic activity and is also realized through the modulation of NHE3 [23].

The potential of histamine as a mediator in renal pathophysiology has been largely disregarded in the scientific community. However, a substantial body of literature supports the hypothesis that histamine is an autacoid playing an active role in renal physiology [24, 25]. The kidney possesses a complete histaminergic machinery, spanning from the enzyme involved in its production (histidine decarboxylase, HDC), to the enzymes responsible for its degradation (diamine oxidase DAO, and histamine-N-methyltransferase HNMT). The hypothesis that an intrarenal production of histamine occurs is supported by the presence of HDC in the kidney. Accordingly, physiological renal levels higher than the circulating once (2–5 pmol/mg organ weight in the kidney and 10 nM circulating) have been reported [26–28]. Furthermore, the likelihood of mast cells contributing to elevated histamine levels within this physiological concentration range is minimal, given their scarcity within healthy kidneys. However, the potential for mast cell contribution to heightened histamine renal levels in disease cannot be discounted.

The locally produced histamine has been shown to activate all four receptor subtypes identified to date. Although, Chen, et al. [29] did not detect the histamine H_1_ and H_4_ receptors in their experimental model, the presence of histamine receptors, albeit at a low level, has been confirmed by the Kidney KIT Project [30–32], Kidney Systems Biology Project [33], and proteomic Human Protein Atlas databases. The observed discrepancies between the studies may be attributable to interspecies variation, as evidenced in the case of the histamine H_4_ receptor [34] and/or the presence of distinct splice variants [35].

We previously demonstrated the renal expression of the histamine receptors ex vivo in humans and animals and in vitro on human cells, including the human kidney (HK)-2 cell line, by immunohistochemistry and pharmacological approaches [36]. Furthermore, all histamine receptors are differentially expressed along the nephron [24, 37], and at least the histamine H_4_ receptor expression increases in the diabetic kidney [38, 39]. The results of our study on the renal expression of histamine receptors have been recently corroborated by Spires et al. [40].

Despite the high levels of histamine present in the kidneys, there is a paucity of evidence regarding its function in this organ. The majority of these have been linked to the vasoactive properties of histamine, but other potential effects cannot be discounted [37, 38]. The only physiological role suggested for histamine is during embryonic development [41], with most of the studies exploring the amine function in the kidney after its exogenous administration. Consequently, distinguishing between the extrarenal, circulating, and locally produced effects of histamine remains a challenging endeavour. Irrespective of its provenance, histamine renal levels have been shown to increase in a number of pathological conditions, including heart failure resulting from acute myocardial infarction, diabetes, renal reperfusion injury, and chronic kidney disease [25, 38, 39, 42–44]. In a murine model of streptozotocin-induced diabetic nephropathy, the blockade of the histamine H_4_ receptor by JNJ39758979 (100 mg/kg/day) [45] and the blockade of the histamine H_1_ receptor by bilastine (30 mg/kg/day) [46] have been shown to reduce renal damage induced by diabetes. The two drugs exerted differential effects, especially at the proximal tubule level. Only JNJ39758979 prevented the reabsorptive machinery imbalance caused by diabetes, preserving the basal expression levels of both megalin and NHE3 [45]. Concurrently, mice demonstrated a recovery in urinary pH and albumin reabsorption. H_4_^−/−^ mice consistently exhibited a reduced basal expression of NHE3 [47]. The studies found no significant impact on hyperglycemia [45–47].

Therefore, we hypothesize that histamine, by activating the histamine H_4_ receptor pathway, may influence NHE3 expression with a direct tubular effect. The objective of the present study was to examine the possible involvement of histamine and its receptors in the functional expression of NHE3 in the HK-2 cell line.

## Materials and methods

### Materials

All products, unless otherwise indicated, including the rabbit polyclonal anti-β-actin antibody (A2066), were acquired from Sigma Aldrich (St. Louis, MO, USA), while the plastic devices, except for the 96-well Assay Black Plate clear-bottom plates (CSL- 3603) from Corning Life Sciences (Lowell, MA, USA), were sourced from Euroclone (Milan, IT). The immortalized proximal tubule epithelial cell line from normal adult human kidney HK-2 (CRL2190TM) was purchased from the American Type Culture Collection (Number CRL-2190). DMEM/F12 w/o glutamine medium (AU-L0094-500), Trypsin–EDTA 1 × in PBS (AU- L0940), and bovine serum (FCS; AU-S1810) as well as the blue x Ray Film came from Aurogene (Rome, IT). L-glutamine (17-605E) and the penicillin–streptomycin mixture (09–757F) were obtained from Lonza Group Ltd (Allendale, NJ, USA). The CellMask™ Orange plasma membrane stain, Alexa-Conjugated secondary antibody donkey anti-Mouse IgG (A-31570), the anti-fade DAPI mounting medium Fluoroshield™ with DAPI, and the BCA™ Protein Assay Kit, BCECF-AM (B1150) were purchased from Thermo Fisher Scientific Inc. (Rockford, IL, USA). The antibody mouse monoclonal anti-NHE3 antibody (sc-136368) was from Santa Cruz Biotechnology (Dallas, TX, USA). The Alexa-Conjugated secondary antibody (115–545-003 Jackson ImmunoResearch, Ely, UK). The Direct-zol RNA extraction kit was purchased from Zimo research (Irvine, CA, USA), while the SensiFAST™ cDNA Synthesis Kit and the SensiFAST SYBR No-Rox Kit were from Bioline (Milan, IT). The Hs_glyceraldehyde-3-phosphate dehydrogenase (GAPDH)_2_SG QuantiTect Primer Assay (QT01192646) and the Hs-SLC9A3_1_SG QuantiTect Primer Assay (QT00066801) were from Qiagen (Hilden, DE). Acrylamide/Bis 29:1 and Skim milk powder for blotting (4259.01) were produced by Serva (Heidelberg, Carl Benz). The Opti-Protein XL Marker molecular weights were from Applied Biological Materials Inc. (Richmond, BC, CDN). The Immobilon® PVDF transfer membrane was from Merck Millipore (Milan, IT). The primary monoclonal mouse anti-phospho (ph)-p38 MAPK (Thr180/Tyr182; 28B10; #9216), anti-ph-ERK1/2 (Thr202/Tyr204; E10; #9106), and the secondary antibodies, sheep anti-rabbit and horse anti-mouse, conjugated to horseradish peroxidase, were purchased from Cell Signaling Technology, Inc. (Danvers, MA, USA). The chemiluminescence identification kit WesternBright™ Quantum was from Advansta (Menlo Park, CA, USA) Clarity MaxTM Western ECL Substrate (1,705,062) was from Bio-Rad Laboratories, Inc. (Hercules, CA, USA). The developing liquid and fixative were from Agfa-Gevaert (Mortsel, BE). Histamine ELISA kit was from ImmunoSol SAS (Talence, France).

Histamine dihydrochloride (PubChem CID 5818), chlorpheniramine maleate (PubChem CID 5281068), JNJ-7777120 (PubChem CID 4908365), SB202190 (PubChem CID 5169), PD98059 (PubChem CID 4713), SP600125 (PubChem CID 8515) and S3226 (PubChem CID 137313644) were dissolved in dimethyl sulfoxide and the final drug concentrations were obtained by diluting the stock solution to perform the experiments; the organic solvent thus obtained had a final concentration of less than 0.1% and, consequently, does not affect cell viability.

### Methods

#### Cell cultures

The HK-2 cells were cultivated in DMEM/F12 medium containing 1 mg/l glucose, supplemented with 10% foetal calf serum (FCS), penicillin (100 IU/ml), and L-glutamine. The cultures were maintained at 37 °C in a 95% air, 5% CO_2_ humidified incubator and subcultured at subconfluence (80%) to avoid phenotypic variations [48] as per the ATCC Product Sheet.

#### Cell treatments

HK-2 at 80% of confluence were exposed to vehicle alone or pretreated for 30 min with chlorphenamine maleate (a selective histamine H_1_ receptor antagonist, 10 µM), JNJ-7777120 (a prototype of the histamine H_4_ receptor antagonists, 1 µM), and challenged with histamine in the range of 0–1000 nM for 0–48 h. The role of MAPKs as signalling molecules has been examined by exposing cells to histamine at a concentration of 100 nM for 0 to 90 min. Furthermore, the cells were pretreated for 30 min with the MAPKs selective inhibitors SB202190 (p38 MAPK inhibitor), PD98059 (ERK1/2 inhibitor), or SP600125 (SAPK/JNK inhibitor), all at 20 µM, before being challenged with histamine (100 nM) for 60 min or 48 h.

#### Immunofluorescence

The HK-2 cells were plated at 80% confluence on collagen-coated cover glasses. They were then stained with CellMask™ Orange plasma membrane stain according to the manufacturer’s protocol. The cells were then fixed with 4% paraformaldehyde for 10 min at RT. Following permeabilization by a solution of PBS (phosphate buffered saline)-triton X (0.1%), the unspecific binding sites were blocked using a solution of 1.5% BSA (bovine serum albumin) in PBS-triton X (0.1% v/v). Samples were subjected to an overnight incubation with anti-NHE3 (1:100 in PBS 0.1% BSA) at 4 °C, followed by incubation with the Alexa-conjugated secondary antibody (Thermo Fisher Scientific Inc). The nuclei were stained with DAPI. A thorough examination of all the slides was conducted at × 63 magnification using the Leica DMI4000 B microscope, with five images captured for each section. Three independent experiments were conducted. To evaluate the effect of histamine on NHE3 expression on the cell membrane, cells plated at 80% confluence on collagen-coated cover glasses were treated with histamine (100 nM) for 0–90 min. At the designated time point, the cells were stained with the CellMask™ Orange plasma membrane stain according to the manufacturer’s protocol and fixed with 4% paraformaldehyde for 10 min at RT. The cell monolayers were subjected to gentle permeabilization with PBS-triton X (0.05% v/v) for 10 min before an unspecific binding blockade by a PBS-1.5% BSA solution. Samples were subjected to an initial incubation with anti-NHE3 antibody at + 4 °C overnight and subsequently with the Alexa-conjugated secondary antibody (Jackson ImmunoResearch). Coverslips were mounted with Fluoroshield™ with DAPI. The observation and acquisition of immunolabelled cells were performed using the Leica DMI4000 B microscope equipped with the 63 × objective lens.

#### RT-qPCR

Total RNA was extracted and isolated from HK-2 cells with the Direct-zol RNA kit according to the manufacturer’s instructions. The quantification of the isolated RNA was determined with the NanoDrop 2000 (Thermo Fisher Scientific). Subsequently, 1 ng of each sample was reverse-transcribed using the SensiFAST™ cDNA Synthesis Kit. Subsequently, 0.5 µL of cDNA was subjected to RT-PCR reaction using the SensiFAST SYBR No-Rox Kit, following the manufacturer’s protocol. The QuantiTect Primer Assay (Qiagen) was utilised as the gene-specific primer pair for SLC9A3. The transcript of the reference gene GAPDH was utilised to normalize mRNA data.

All samples were analysed in duplicate, and at least two no-template controls were included in all RT-PCR runs. In these, 0.5 µL of RNase-free water was added to the reaction mix instead of cDNA. The RT-PCR reaction was conducted using the MiniOpticon Real-Time PCR (BIO-RAD, Milan), following the following protocol conditions: the temperature was increased to 95 °C for 2 min, then maintained at this temperature for 5 s. This was followed by a 10 s period at 60 °C, and finally a 13 s period at 72 °C. This sequence was repeated for a total of 40 cycles. In all PCR runs, a minimum of three independent cDNA preparations per sample were utilised, in conjunction with a duplicate run for all samples, incorporating two non-template controls. The results were analysed using the 2^−ΔΔCt^ method to determine relative expression.

#### Immunoblotting

Twenty μg of protein extracted from HK-2 by ice-cold buffer (Tris/HCl 10 mM pH 7.4, NaCl 10 mM, MgCl_2_ 1.5 mM, 1% triton X), with the addition of Sigmafast, protease inhibitor mixture (PIC 1:1000), phenylmethyl sulphonyl fluoride (PMSF, serine protease inhibitor) 1 mM, sodium fluoride (Na_2_F) 1 mM, and sodium orthovanadate 1 mM, were subjected to SDS-PAGE using a 8 or 10% gel. The proteins were transferred onto a PVDF membrane at a constant voltage of 100 V for 60 min. Following the blocking of non-specific binding sites with 5% milk in PBS-Tween 1 × for 1 h at room temperature, the PVDF membrane was blotted overnight at 4 °C with NHE3 (1:200), ph-p38 MAPK (1:1000), or ph-ERK1/2 (1:2000) antibodies. β-actin (1:10,000) was utilised as a loading uniformity control protein. The respective secondary antibodies were used at a concentration of 1:3000 and incubated for 1 h at room temperature. The membranes were overlaid with WesternBright™ Quantum and subsequently exposed to either Hyperfilm ECL or Clarity Max Western ECL. Visualisation was then undertaken using the ChemiDoc Touch BioRad Imaging System. The densitometric analysis was performed on the digitized images by the ImageJ software package. The optical density (OD) values were derived from the background-corrected band intensity and band area of the targeted protein. The normalisation process entailed the division of the observed O.D. (optical density) values of the target protein by the O.D. value of the reference β-actin.

#### NHE3 activity assay

The measurement of NHE3 activity was conducted using a fluorometric approach, employing the ratiometric method. This method is predicated on the utilisation of a fluorescent pH-sensitive indicator, 2′,7′-Bis-(2-carboxyethyl)-5-(6)-carboxyfluorescein, acetoxymethyl ester (BCECF-AM). The cells were seeded on a black, clear-bottom 96-well plate and loaded with 5 μM BCECF-AM (40 min at 37 °C) in Hank’s Balanced Salt Solution (HBSS) with 20 mM HEPES (Supplementary material, Table S_1). Intracellular pH (ipH) was estimated from the ratio of fluorescence intensity measured at 490 nm or 440 nm and fixed emission at 535 nm by the EnSight® multimode plate reader (PerkinElmer Inc., Waltham, MA, USA). The 500/450-nm fluorescence ratio was calibrated to ipH via the K^+^/nigericin method (8-point curve in the pH range 6.2 – 7.6). Following basal recording, cells were loaded with 20 mM NH_4_Cl to induce cytoplasm alkalisation for NH_3_ formation. The alterations induced by NH₄Cl loading in the absence of HCO_3_^−^ were observed over 5 min. The recovery phase was induced by replacing the NH_4_Cl buffer with HCO_3_^−^-free HBSS with 20 mM HEPES buffer (Supplementary material, Table S_1). This process was monitored for 15 min, or until a plateau was reached. The rate of ipH recovery after the acid load, representing the measure of NHE3 activity, was measured in terms of the initial rate of pH recovery (ΔipH/dt, pH units/min) from the first 2 min after the start of the recovery phase by linear regression analysis performed by the Prism 10 program (GraphPad Software, Inc., San Diego, CA, USA). The NHE3 selective inhibitor S3226 (100 nM, 30 min) was used as an internal control. All experiments were conducted in triplicate, and the results are the mean of at least five separate experiments.

#### Histamine production by HK-2 growing cells

The histamine concentrations in the medium from HK-2 growing cells were determined by ELISA assay according to the manufacturer’s instructions. In summary, 20 μL of the medium from cells seeded on a 96-well plate was collected when the culture reached 80% confluence. The collected medium was then exposed to the diluent provided by the kit before the addition of the acylation reagent and buffer for 15 min. Concurrently, the standards, controls, and unconditioned DMEM/F12 medium supplemented with 10% FCS were subjected to the same protocol. Subsequently, 25 µL of the obtained samples were processed by ELISA.

#### Statistical analysis

The data are reported as the mean ± standard error of the mean (SEM). The normality of the distribution of the results was checked by the Kolmogorov–Smirnov test. Significant differences between two individual means were determined by the unpaired Student’s t-test in case of normality distribution. When normality distribution was not satisfied, the Mann Whitney test was used to determine significant differences between two individual means. In case of more than two groups of treatment, a one-way analysis of variance (ANOVA) followed by Dunnett’s post-hoc test was used to assess significant differences between each treatment group and the control or S3226, when the data were not normally distributed, the non-parametric test of Kruskal–Wallis followed by Dunn’s multiple comparison test was used to assess significant differences between each treatment group and the control. The determination of the best-fitting four-parameter logistic equation was undertaken to produce the concentration–response curve. The probability value (*P*) ≤ 0.05 was selected as the cut-off for statistical significance.

## Results

### HK-2 cells basal expression of NHE3

**Fig. 1:**
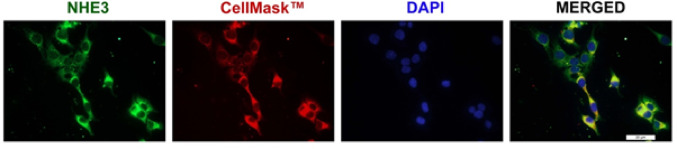
Basal expression of NHE3 in the HK-2 cell line. Representative image of immunofluorescence analysis of the expression of NHE3 and its partial localization in the membrane. NHE3 was detected by conjugation with the specific antibody anti-NHE3 (green). The CellMask™ Plasma Membrane Stains (red) highlighted the cell membrane, while the nuclei were stained with DAPI (blue). Photographs were obtained using the Leica DMI4000B automated inverted microscope equipped with a 63 × objective lens. Scale bar: 20 μm

To validate the in vitro model of HK-2 cells for studying NHE3, its expression was confirmed by immunofluorescence (Fig. 1).

**Fig. 2:**
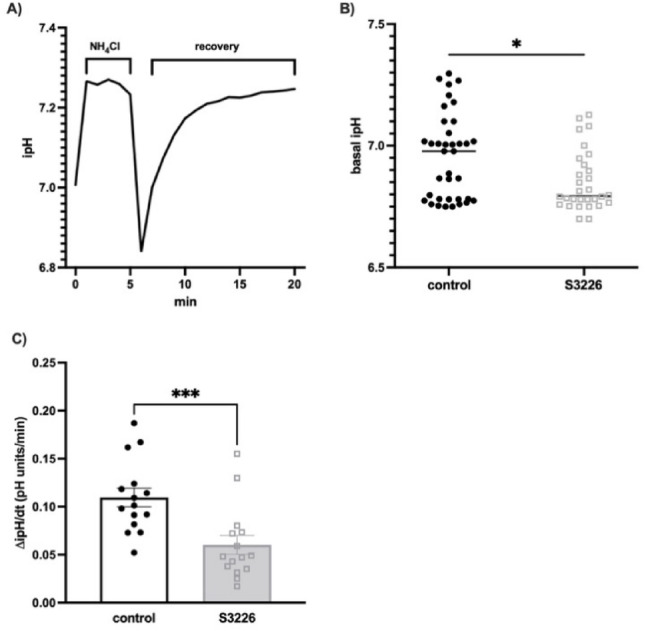
NHE3 basal activity in HK-2 cell line. **A** The ipH change was obtained by challenging HK-2 cells with NH_4_Cl 20 mM for a 5 min period, followed by replacement with HCO_3_^−^–free HBSS (recovery phase) for 15 min. **B** Median effect of the NHE3 inhibitor S3226 100 nM for 30 min on the HK-2 basal ipH (each dot represents one well of HK-2 cells at 80% confluency); a statistically significant difference was found via the Mann Whitney test; ^*^ *P* ≤ 0.05. C rate of the ipH recovery phase (ΔipH/dt) in HK-2 cells without or with S3226 100 nM for 30 min; a statistically significant difference was found via the Mann Whitney test; ^***^P ≤ 0.001. Experiments are the mean ± SEM of at least 5 independent experiments run in triplicate

Probing cells with the anti-NHE3 antibody produced robust staining predominantly on the cell membrane, as highlighted by the partial overlay with the CellMask™ plasma membrane stain. The NHE3 expression was also functionally validated by the classical ammonium pre-pulse technique to measure NHE3 activity (Fig. 2A). The NH_4_Cl loading resulted in a rapid intracellular alkalinization, followed by a fall in ipH from 7.23 ± 0.02 to 6.84 ± 0.04 (*P* < 0.0001) upon NH_4_Cl removal, before the beginning of recovery due to the intracellular alkalization driven by NHE3 through H^+^ extrusion. The NHE3 dependency of the observed phenomenon was confirmed by challenging cells for 30 min with the NHE3 selective inhibitor S3226 at 100 nM. As demonstrated in Fig. 2B and 2C, S3226 affected the basal ipH (from 7.14 ± 0.04 to 6.99 ± 0.04; *P* ≤ 0.05), and reduced the ΔipH/dt of the recovery phase (*P* ≤ 0.05; Fig. 2C).

### Effect of histamine on gene and protein expression of NHE3

**Fig. 3:**
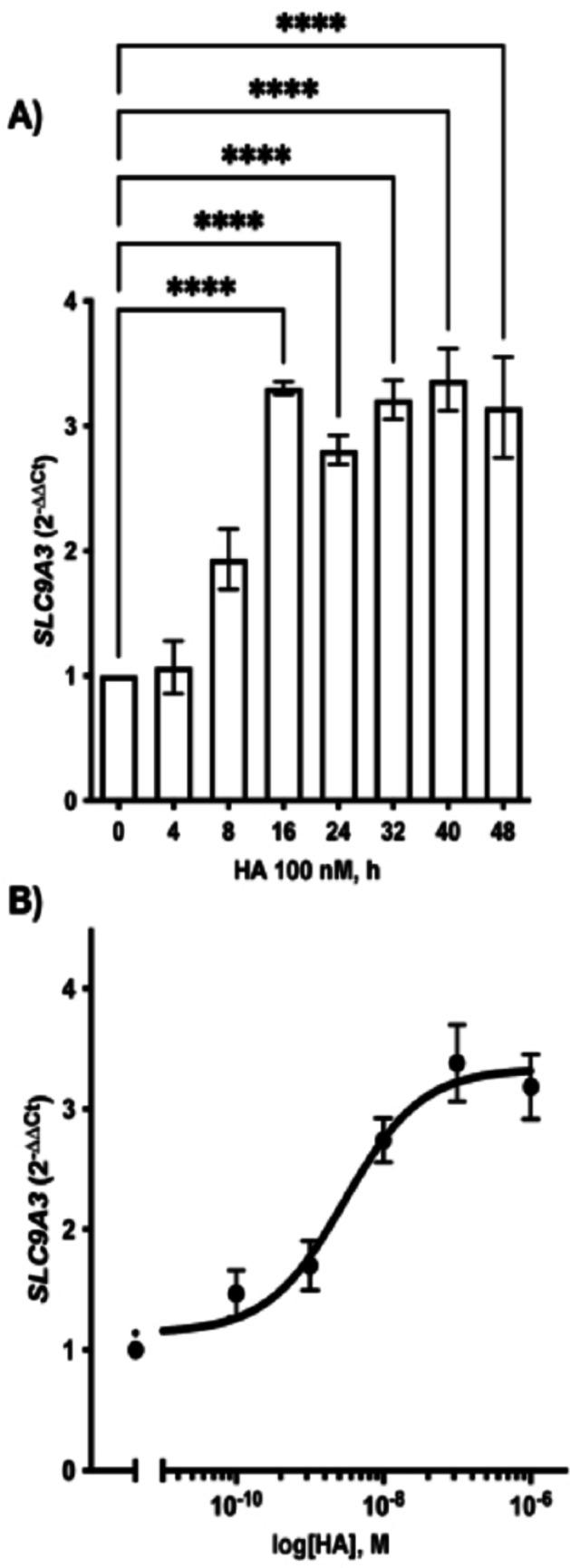
Effect of histamine on the expression of SLC9A3. **A** Analysis of *SLC9A3* mRNA expression in HK-2 cells exposed to additional 100 nM histamine (HA) for 0–48 h; ^****^ *P* ≤ 0.0001: statistically significant *vs* untreated condition determined via Kruskal–Wallis followed by Dunn’s multiple comparison test. **B** Best fitting of the concentration-curve response of *SLC9A3* expression in HK-2 cells exposed to additional 0–1000 nM histamine (HA) for 24 h. The results were analyzed using the 2^−∆∆Ct^ method and expressed as mean ± SEM of 4 independent experiments, run in duplicate

Previous in vivo data suggest that histamine, binding to the histamine H_4_ receptor, has a causal effect on NHE3 overexpression in diabetic mice [45, 47]. Thus, we challenged HK-2 cells with histamine, by considering a range of added histamine 0–1000 nM for 0–48 h and by evaluating the mRNA expression of *SLC9A3*, the gene encoding for NHE3, by RT-PCR. As shown in Fig. 3, histamine induces *SLC9A3* expression in a time- and concentration-dependent manner with a maximum after adding 100 nM for 16 h. Furthermore, the histamine effect on *SLC9A3* gene expression was still constant up to 48 h.

**Fig. 4:**
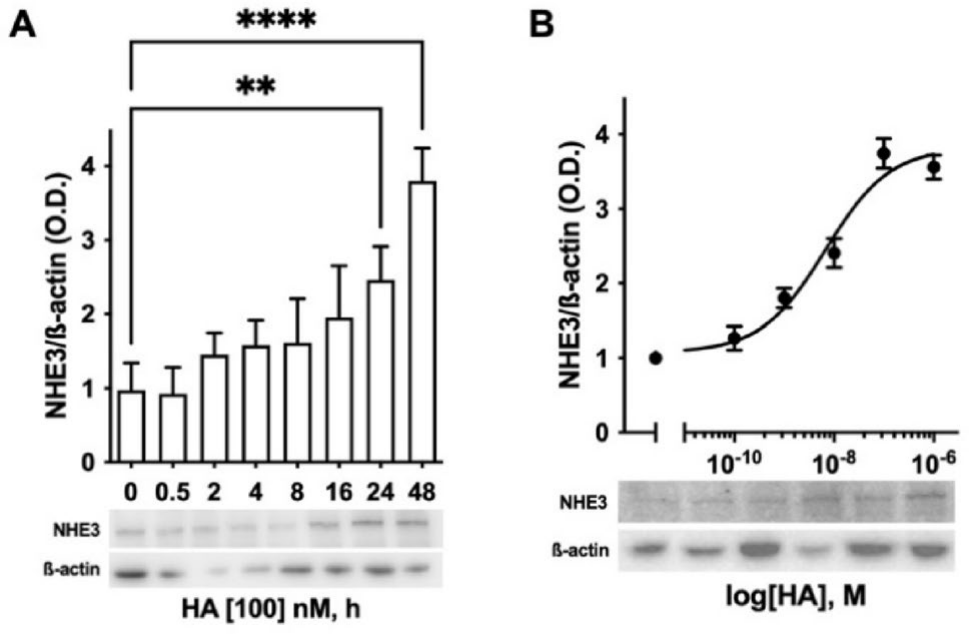
Effect of histamine on NHE3 protein expression. **A** Densitometric analysis of NHE3 protein expression in HK-2 cells exposed to additional 100 nM histamine (HA) for 0–48 h. The results, expressed as optical density (O.D.), are the mean ± SEM of 3 independent experiments normalized for β-actin. ^**^ *P* ≤ 0.01; ^****^ *P* ≤ 0.0001: statistical significance *vs* untreated condition, determined via ANOVA followed by Dunnett’s post-hoc test. The radiograph image is representative of immunoblotting bands corresponding to the expression of NHE3 and the relative β-actin band, used as a reference protein. **B** Best fitting of concentration-curves response of NHE3 expression in HK-2 cells exposed to additional 0–1000 nM histamine (HA) for 48 h. The results, expressed as optical density (O.D.), are the mean ± SEM of 3 independent experiments normalized for β-actin

The effect of histamine on NHE3 was also investigated at the protein level, after validating the NHE3 antibody (Supplementary material Fig. S_1), with corroborating results. Histamine induced the overexpression of NHE3 in a time- and concentration-dependent manner (Fig. 4), with a maximum in protein overexpression after adding 100 nM histamine for 48 h.

### Effect of histamine on NHE3 activity

**Fig. 5:**
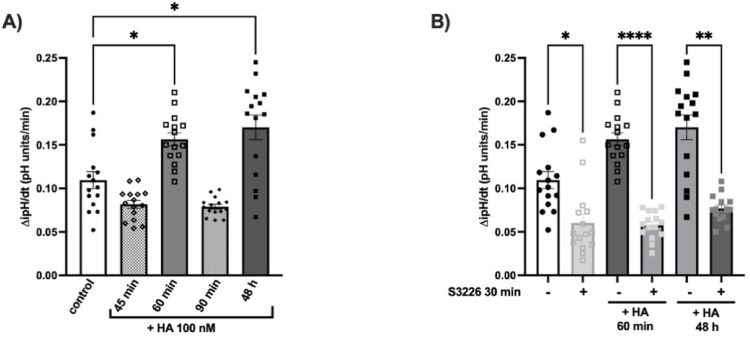
Effect of histamine 100 nM on NHE3 activity in the HK-2 cell line. **A** Rate of the ipH recovery phase (ΔipH/dt) in HK-2 cells exposed to additional 100 nM histamine (HA) for 45 min, 60 min, 90 min, or 48 h; ^*^ *P* ≤ 0.05 *vs* control determined via Kruskal–Wallis followed by Dunn’s multiple comparison test. Experiments are the mean ± SEM of 5 independent experiments run in triplicate. **B** Rate of the ipH recovery phase in HK-2 cells after pretreatment with S3226 100 nM for 30 min, followed by the addition of 100 nM histamine (HA) for either 60 min or 48 h. ^*^ *P* ≤ 0.05, ^**^ *P* ≤ 0.01 ^****^ *P* ≤ 0.0001 *vs* SB3226 determined via Kruskal–Wallis followed by Dunn’s multiple comparison test. Experiments are the mean ± SEM of 5 independent experiments run in triplicate

To evaluate whether the effect of histamine could also induce a change in NHE3 activity, the ipH variations evoked by histamine 100 nM for 0–48 h were measured. As demonstrated in Fig. 5A, the administration of histamine resulted in an increase in the ΔipH/dt after 60 min (*P* ≤ 0.05). The phenomenon appears to be transient, as no measurable changes were observed in the recovery phase after 90 min. Notably, consistent with NHE3 gene and protein expressions, the activity of NHE3 is again increased after 48 h of histamine challenge (*P* ≤ 0.01 *vs* control; Fig. 5A).

**Fig. 6:**
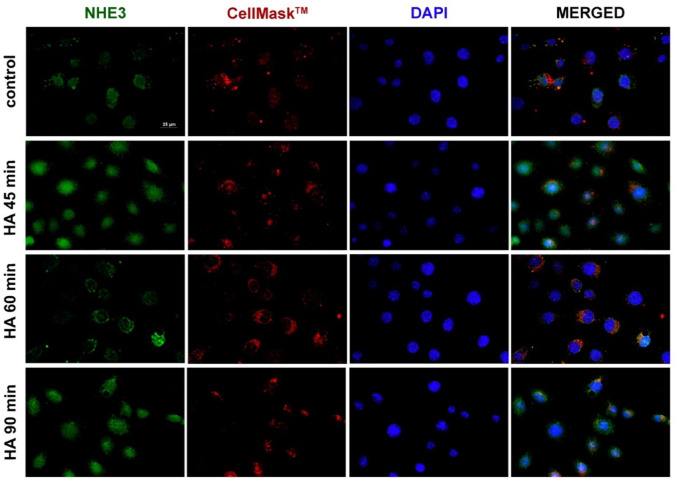
Basal expression of NHE3 in the HK-2 cell line. Representative image of immunofluorescence analysis of the expression of NHE3 and its partial localization in the membrane in HK-2 cells challenged with additional 100 nM histamine (HA) for 45 min, 60 min or 90 min. NHE3 staining was obtained after conjugation with the specific antibody anti-NHE3 (green). The CellMask™ Plasma Membrane Stains (red) highlighted the cell membrane, while the nuclei were stained with DAPI (blue). Acquisition was performed using the Leica DMI4000B automated inverted microscope equipped with a 63 × objective lens. Scale bar: 25 μm

The immunofluorescence analysis demonstrated that histamine 100 nM induced a more pronounced membrane localisation of NHE3 after 60 min, as evidenced by its overlay with CellMask™ plasma membrane stain, followed by the NHE3 internalisation at 90 min (Fig. 6). Finally, the effect evoked by histamine 100 nM at either 60 min or 48 h on NHE3 was blunted when cells were pretreated with S3226 100 nM for 30 min before the histamine challenge (Fig. 5B), confirming that the changes in ΔipH/dt values induced by histamine were NHE3 dependent.

### Effect of chlorphenamine and JNJ-7777120 on NHE3 expression

**Fig. 7:**
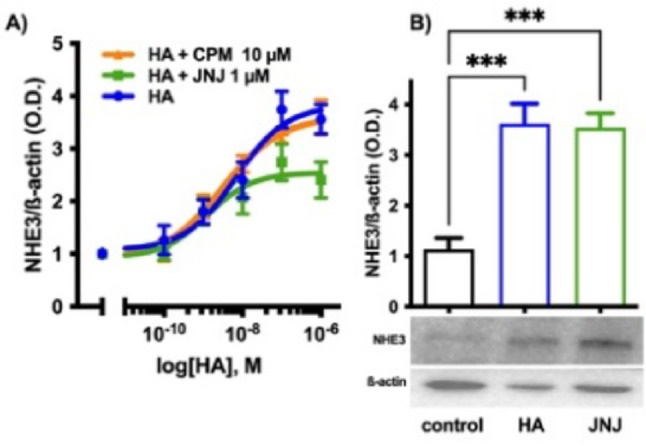
Effect of chlorphenamine 10 µM and JNJ-7777120 1 µM on NHE3 expression. **A** Best fitting of concentration-curves response of NHE3 expression in HK-2 cells exposed to additional 0–1000 nM histamine (HA) for 48 h, alone or in the presence of chlorphenamine (CPM) 10 µM or JNJ-7777120 (JNJ) 1 μM. **B** Densitometric analysis of treatment-induced NHE3 expression with additional 100 nM histamine (HA) or JNJ-7777120 (JNJ) 1 µM for 48 h. The results, expressed as optical density (O.D.), are the mean ± SEM of 3 independent experiments normalized for β-actin. ^***^ *P* ≤ 0.001 determined via ANOVA followed by Dunnett’s post-hoc test. The radiograph image is representative of immunoblotting bands corresponding to the expression of NHE3, and the relative β-actin band is used as a control protein

To dissect how the histamine H_1_ and H_4_ receptors contribute to histamine-induced NHE3 expression, cells were exposed to histamine 0–1000 nM alone or in the presence of chlorphenamine (histamine H_1_ receptor antagonist) 10 µM or JNJ-7777120 (histamine H_4_ receptor antagonist prototype) 1 µM, after excluding any effect on cell viability (Supplementary material Fig. S_2). As shown in Fig. 7A, only JNJ-7777120 affects the histamine concentration–response curve, reducing the maximum effect reached. JNJ-7777120 alone induced the expression of NHE3 to the same extent as histamine 100 nM (Fig. 7B), confirming its partial agonist behavior (or pharmacodynamic profile) [49].

**Fig. 8:**
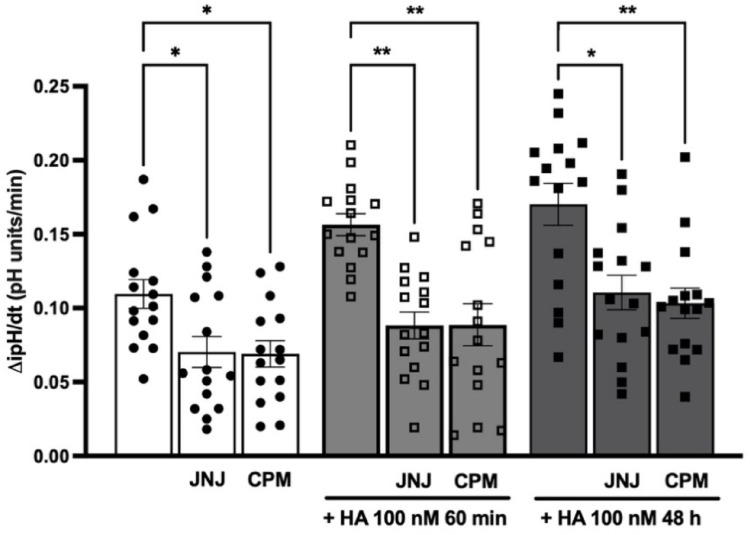
Effect of chlorphenamine 10 µM and JNJ-7777120 1 µM on NHE3 activity. Rate of the ipH recovery phase (ΔipH/dt) in HK-2 cells treated with chlorphenamine (CPM) 10 µM and JNJ-7777120 (JNJ) 1 µM for 30 min before the challenge with additional 100 nM histamine (HA) for 60 min or 48 h; ^*^ *P* ≤ 0.05 and ^**^ *P* ≤ 0.01 determined via Kruskal–Wallis followed by Dunn’s multiple comparison test. Experiments are the mean ± SEM of 5 independent experiments run in triplicate

However, unexpectedly, when the effect on NHE3 activity was measured, both histamine H_1_ and H_4_ receptors appeared involved. Indeed, both chlorphenamine 10 µM and JNJ-7777120 1 µM reduced the ΔipH/dt to a comparable extent at the basal condition and after challenging with histamine 100 nM for 60 min or 48 h (Fig. 8).

To further investigate the basal activity of the two histamine receptors on NHE3, we measured the concentration of histamine in the medium from growing cells. The data reported in Table 1 confirm that the DMEM/F12 supplemented with 10% FCS already contains histamine and that HK-2 cells produce and release histamine.

**Table 1:** Histamine content in the grown medium

	nM
DMEM/F12 supplemented with 10% FCS	69.39 ± 6.36
HK-2 conditioned medium	157.40 ± 12.16^***^

^***^P ≤ 0.001 *vs* DMEM/F12 supplemented with 10% FCS determined by the unpaired Student’s t-test. Results are the mean ± SEM of 3 independent experiments run in duplicate

### Involvement of MAPKs in NHE3 expression and activation induced by histamine

**Fig. 9:**
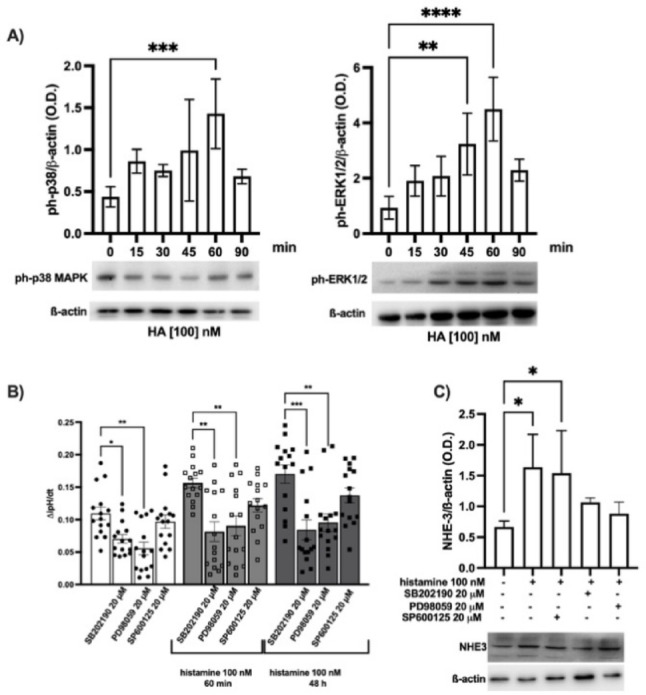
MAPK involvement in the NHE3 regulation elicited by histamine. **A** Densitometric analysis of the effect evoked by additional 100 nM histamine (HA) for 0–90 min on the phosphorylation of p38 MAPK or ERK1/2. The results, expressed as optical density (O.D.), are the mean ± SEM of 3 independent experiments normalized for β-actin. ^**^ *P* ≤ 0.01 and ^***^ *P* ≤ 0.001, and ^****^ *P* ≤ 0.0001 were determined via ANOVA followed by Dunnett’s post-hoc test. The radiograph image is representative of immunoblotting bands corresponding to the expression of ph-p38 MAPK, ph-ERK1/2, and the relative β-actin band, used as a control protein. **B** Rate of the ipH recovery phase (ΔipH/dt) in HK-2 cells after MAPK inhibition by SB202190 (p38 MAPK), PD98059 (ERK1/2), or SP600125 (SAPK/JNK) 20 µM for 30 min before challenging with additional 100 nM histamine for 60 min or 48 h; ^*^ *P* ≤ 0.05 and ^**^ *P* ≤ 0.01; ^***^ *P* ≤ 0.0001 determined via Kruskal–Wallis followed by Dunn’s multiple comparison test. Experiments are the mean ± SEM of 5 independent experiments run in triplicate. **C** Densitometric analysis of the NHE3 expression after MAPK inhibition by SB202190 (p38 MAPK), PD98059 (ERK1/2), or SP600125 (SAPK/JNK) 20 µM for 30 min before challenging with additional 100 nM histamine for 48 h. The results, expressed as optical density (O.D.), are the mean ± SEM of 3 independent experiments normalized for β-actin. ^*^ *P* ≤ 0.05 determined via ANOVA followed by Dunnett’s post-hoc test. The radiograph image is representative of immunoblotting bands corresponding to the expression of NHE3, and the relative β-actin band, used as a control protein

As MAPKs are known regulators of NHE3 activity [2, 50], and histamine H_1_ and H_4_ receptors synergically activate p38 MAPK and ERK1/2 pathways [51], we evaluated whether this regulatory pathway could account for the histamine-induced activity of NHE3 in the HK-2 cells. As shown in Fig. 9A, histamine 100 nM induces in a time-dependent manner the phosphorylation of both p38 MAPK and ERK1/2 but not of JNK/SAPK (Supplementary Fig. S_3). This effect is achieved after 45 min for ERK1/2 and 60 min for p38 MAPK, and declines for both at 90 min. The selective inhibition of p38 MAPK by SB202190 or ERK1/2 by PD98059 (both at 20 µM) prevented the increase induced by histamine of both the NHE3 activity (Fig. 9B) and protein expression (Fig. 9C).

## Discussion

The results of this study demonstrate, for the first time, that histamine directly induces the expression and the activity of NHE3 in the proximal tubule cells. The kinetics of the NHE3 activation by histamine is biphasic, with the amine inducing the NHE3 activity through early (60 min) phosphorylative and late (48 h) transcriptional events. These findings are consistent with the ability of the amine to evoke acute and transient effects (within seconds to minutes), like the rapid increase in endothelial permeability [52–56], or delayed effects (within hours), like the reduction of junctional proteins [57, 58].

Different literature evidence points to histamine as an autacoid locally produced in the kidney [24]. However, apart from its potential role during kidney organogenesis [41], the physiological role of histamine in the kidney has been scantly investigated, with most of the evidence coming from models of renal diseases. We have previously demonstrated that the histamine H_4_ receptor is involved in NHE3 expression, assessing the effect of JNJ39758979 in a model of diabetic nephropathy [45]. The present in vitro study demonstrates, for the first time, the direct effects of histamine on proximal tubular cells, and more specifically, on NHE3.

The cellular model we selected was the HK-2 cells because extensively used as an experimental model in translational research [59], although these cells do not accurately represent normal human proximal tubule physiology. In particular, Khundmiri, et al. [60] reported that HK-2 cells express only 25.9% of the proximal tubule-specific markers. These findings have prompted a re-evaluation of NHE3 expression in HK-2 cells. However, accumulating evidence documented the expression of NHE3 in these cells [61–67]. The contrasting data on NHE3 expression could find an explanation in the cell-growth conditions, which can affect the HK-2 cell phenotype. In the study by Khundmiri, et al. [60] the confluency was set at 90–95%, but as reported in the ATCC Product Sheet, HK-2 phenotype changes when more than 80% confluence is reached. Furthermore, Garmaa, et al. [68] demonstrated that HK-2 cells exhibit different behavior when the culture medium is changed. Finally, phenotype changes could also occur during the passaging process [48]. Herein, by immunofluorescence, immunoblotting, and functional assay, we confirmed that HK-2 cells grown at 80% confluence in DMEM/F12 medium supplemented with 10% FCS, basally express NHE3, which is constitutively active. Therefore, despite the limitations of this cell line in recapitulating all the features of normal human proximal tubule, in our experimental setting HK-2 cells are still a useful tool. More importantly, the HK-2 cells represent a suitable tool also to study the renal histaminergic system since: (i) they constitutively express two out of four histamine receptors, i.e. H_1_ and H_4_ receptors [36]; (ii) the growth medium for HK-2 cells has already 0.15 mM of L-histidine, that is the histamine precursor. Consistently, we found a content of histamine of already 69.39 ± 6.36 nM (7.71 ng/ml) in the medium, possibly due to the presence of HDC in the FCS. Moreover, the increased levels of histamine in the medium of growing cells we measured (157.40 nM = 17.49 ng/ml) confirm the ability of HK-2 to basally produce histamine. The measured concentration of histamine in the growth medium is pharmacologically active, being the pKi for the human histamine H_1_ and H_4_ receptors of 4.7–5.9 and 7.8–8.3, respectively [69]. Here, the involvement of both receptors in the NHE3 activation has been confirmed by the use of histamine H_1_ and H_4_ receptor antagonists. Chlorphenamine and JNJ-7777120 reduced the basal activity of NHE3, affecting the recovery slope. This data is consistent with the presence of histamine in the growing cell medium.

In our experimental condition, the added histamine increased NHE3 expression on the membrane and its activity already at 60 min. The parallel activation of p38 MAPK and ERK1/2 was observed. Based on our data, we propose that histamine acutely affects NHE3, activating p38 MAPK and ERK1/2. Interestingly, the selective p38 MAPK and ERK1/2 inhibitors were already effective on the basal NHE3 activity, and both blunted the effect evoked by histamine. Overall, these data confirm their role in NHE3 phosphorylation/dephosphorylation and the intracellular trafficking already described [2, 50].

Antagonizing histamine H_1_ and H_4_ receptors blunted the effect evoked by histamine at 60 min. Although a concentration–response curve was not performed on ipH variation, by using the two antagonists, chlorphenamine and JNJ-7777120, similar effects have been observed, suggesting again a redundant role of the histamine H_1_ and H_4_ receptors. These receptors have already been shown to cooperatively regulate MAPK activation [51]. Histamine H_1_ receptor is a Gq-protein-coupled receptor canonically associated with IP_3_ production and the consequent mobilization of Ca^2+^ from intracellular stores and downstream activation of PKC [70, 71]. Elevated Ca^2+^ concentration promotes NHE3 translocation to the plasma membrane [72]. PI3K is another mainstream of histamine H_1_ receptor activation [36, 73] that could phosphorylate ezrin, a cytoskeletal cross-linking protein essential for NHE3 translocation [74]. On the other side, the histamine H_4_ receptor is a Gi-coupled receptor with a consequent inhibitory effect on cAMP production. cAMP levels are inversely correlated to NHE3 activation [75, 76]. Therefore, we conclude that histamine H_1_ and H_4_ receptors, via MAPK signaling, are involved in histamine induced NHE3 intracellular trafficking.

Besides the acute and transient effects at 60 min, we measured a late response of NHE3 to histamine stimulation, with the induction of NHE3 protein expression and activity at 48 h. Apart from the intracellular rapidly discharged pool of NHE3, a second pool that moves to the apical membrane with slower kinetics has already been demonstrated [77]. Nevertheless, a gene transcription effect could modulate the NHE3 content [72]. In our setting, the MAPK inhibitors SB202190 and PD98059 affected the NHE3 overexpression and activation induced by histamine at 48 h, and again, chlorphenamine and JNJ-7777120 were both effective in NHE3 activity modulation. However, only JNJ-7777120 prevented the increased expression of NHE3 in a concentration-dependent manner. These data suggest that the histamine H_1_ receptor could be involved in early and late NHE3 membrane trafficking processes, but only the histamine H_4_ receptor modulates gene and protein transcription. Accordingly, the histamine H_4_ receptor is associated with alternative signaling pathways, including the ß-arrestin [78], which could explain the implication of the only histamine H_4_ receptor in the transcriptional events. The effect shown by histamine H_4_ receptor in vitro is in keeping with and could mechanistically explain the protective renal effect we demonstrated in vivo for JNJ39758979 in our previous research [45]. This drug, without affecting hyperglycemia, prevented the onset of hyperfiltration, the increase of the albumin to creatinine ratio (ACR), the reduction of the creatinine clearance, and the lowering of the urinary pH. In parallel, the basal levels of NHE3 in the proximal tubule were preserved [45].

One limitation of the study is that the receptor antagonists and MAPK inhibitors were solely used as experimental tools to confirm that the observed histamine effects were mediated by receptor activation, followed by the phosphorylation of p38 and ERK1/2 MAPKs. However, both histamine receptors and p38 MAPK and ERK1/2 inhibitors were effective in reducing basal NHE3 levels. Although this could be explained by the high basal histamine levels, it cannot be ruled out that the effects they evoked involve the modulation of other effectors, such as NHE1. This is particularly interesting in the case of chlorphenamine, a first-generation antihistamine drug with questionable selectivity. The *K*_i_ value for chlorphenamine at the histamine H_1_ receptor is 7 nM [79], but it also inhibits serotonin uptake and displays anticholinergic activity (*K*_i_ value of 31 nM) [80], with a *K*_i_ value of 20–30 μM for the muscarinic receptor [81]. Therefore, effects apart from histamine H_1_ receptor blockade may occur and modulation of NHE1 cannot be ruled out.

In our study, we found a basal histamine concentration of 150 nM. This level is high compared to the added doses of histamine, but relatively low compared to the amounts identified by Sedor and Abbound in the glomerulus [28]. These discrepancies are difficult to explain and complicate any functional interpretations that could be made based on our data. However, it should be noted that data on renal histamine content is outdated and based on limited observations: the study by Sedor and Abbound was conducted in 1984 on just two samples of human kidney tissue. Therefore, it is impossible to determine the exact amount of histamine present in the kidney, particularly in the proximal tubule. Data obtained from growing cells suggests the presence of nascent histamine, a hypothesis that has also been put forward at the cardiac level [82]. However, the physiological role of nascent histamine remains unclear. Consequently, the intriguing hypothesis that physiologically histamine in the kidneys may subserves to the maintenance of the steady-state plasma Na⁺ levels, to which NHE3 is vital [7, 83] remains speculative.

The effect of histamine when its concentration increases above baseline is still difficult to interpret. Firstly, as previously mentioned, histamine’s effects become evident with relatively small changes in concentration compared to baseline, reaching saturation in terms of NHE3 gene and protein expression with the addition of 100 nM. Furthermore, it is not possible to compare the effective levels of histamine found here with those present in the kidneys under physiological or pathological conditions. All of this raises doubts about the significance of histamine’s role in regulating NHE3. The hypothesis that a rise in renal histamine, observed in conditions like heart failure caused by a heart attack, reperfusion injury, or chronic kidney disease [42–44], including diabetic nephropathy, leads to a harmful increase in NHE3 and heightened hyperfiltration [84], deserves further investigations. However, a first clinical trial for repurposing the histamine H_1_ receptor antagonist fexofenadine demonstrated the efficacy of the drug in reducing the ACR in patients with Type 2 diabetes mellitus (at least from six months) and stage 2 or 3 diabetic nephropathy. Moreover, although the control group (ramipril only) and the fexofenadine group (ramipril plus fexofenadine) showed after six months of treatment comparable mean values of eGFR, its decline from baseline was registered only in the control group [85].

In conclusion, our data support the hypothesis that renal histamine via histamine H_1_ and H_4_ receptors can trigger the p38 MAPK and ERK1/2 phosphorylation, leading to both early (60 min, due to phosphorylation and trafficking) and late (48 h, due to gene and protein over-expression) NHE3 activation.

## Supplementary Information

Below is the link to the electronic supplementary material.

Supplementary Material 1

Supplementary Material 2

Supplementary Material 3

Supplementary Material 4

Supplementary Material 5

Supplementary Material 6

## Data Availability

All data are provided in this study, and raw data can be requested to the corresponding author, original composite images or X-ray films for immunoblotting are available in a separate Supplementary Material 2.
