# Supplementary figures and images for: Histamine regulates the activity and the expression of the Na+/H+ exchanger (NHE)3 in human epithelial HK-2 cells
Source: Inflamm Res. 2025 Sep 12;74(1):122. doi: 10.1007/s00011-025-02095-4 (PMC12432042; doi:10.1007/s00011-025-02095-4)

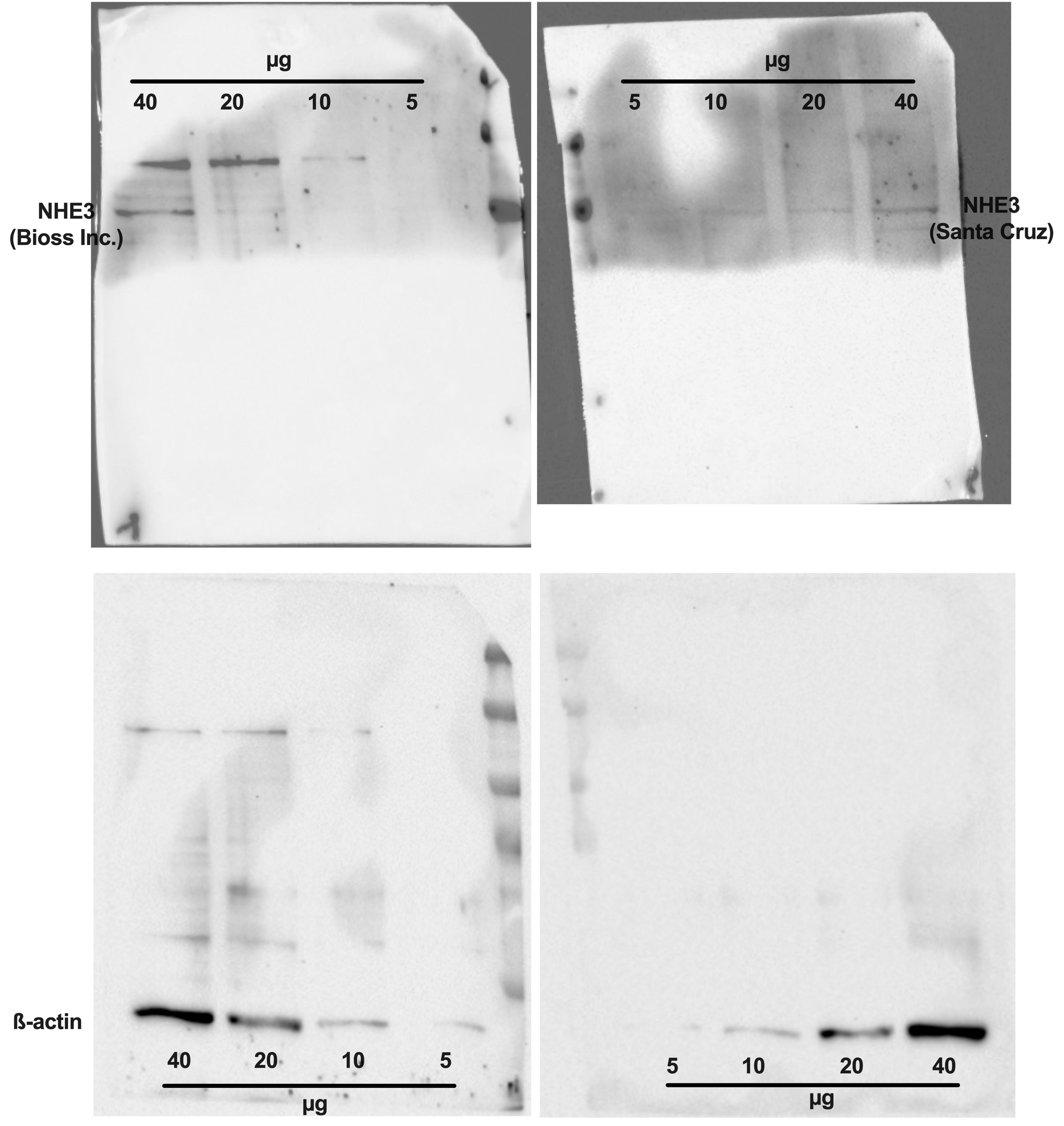

Supplement: Supplementary file 1 — Supplementary Material 1 [file 11_2025_2095_MOESM1_ESM.jpg]

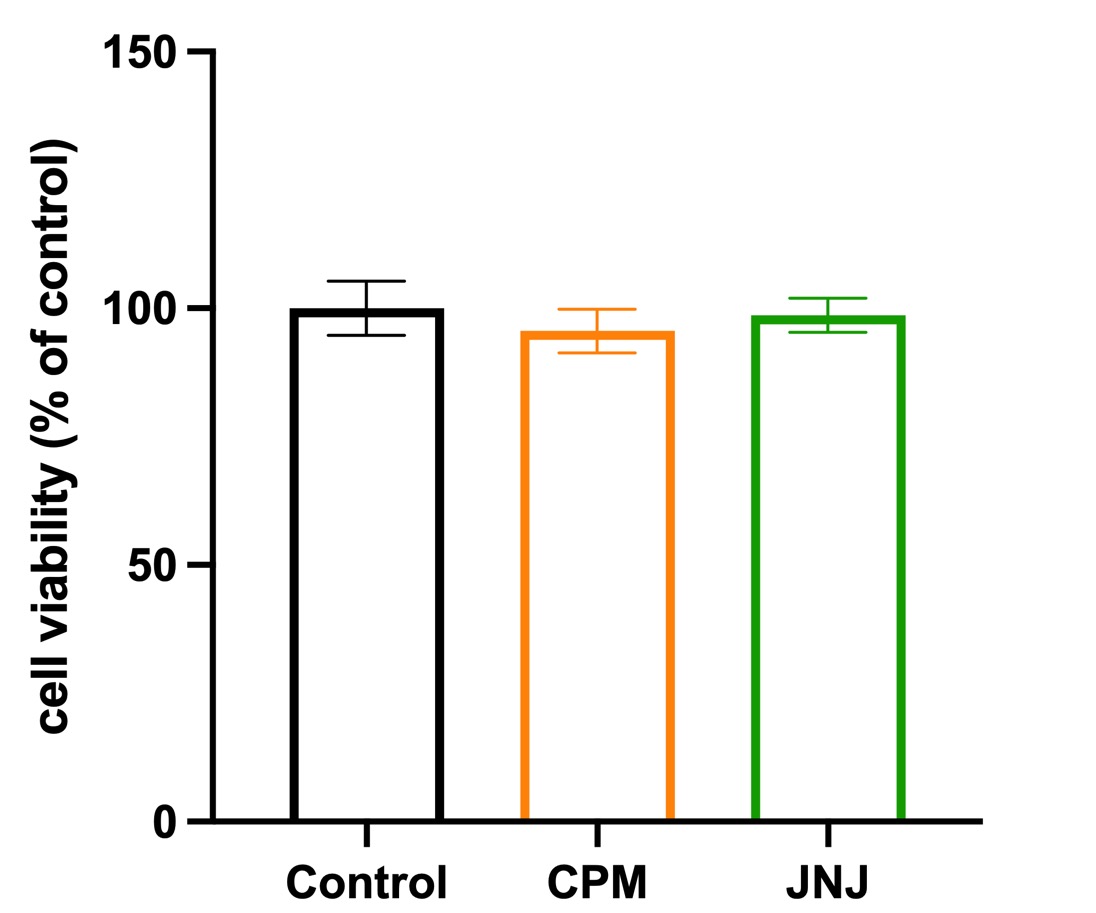

Supplement: Supplementary file 2 — Supplementary Material 2 [file 11_2025_2095_MOESM2_ESM.jpg]

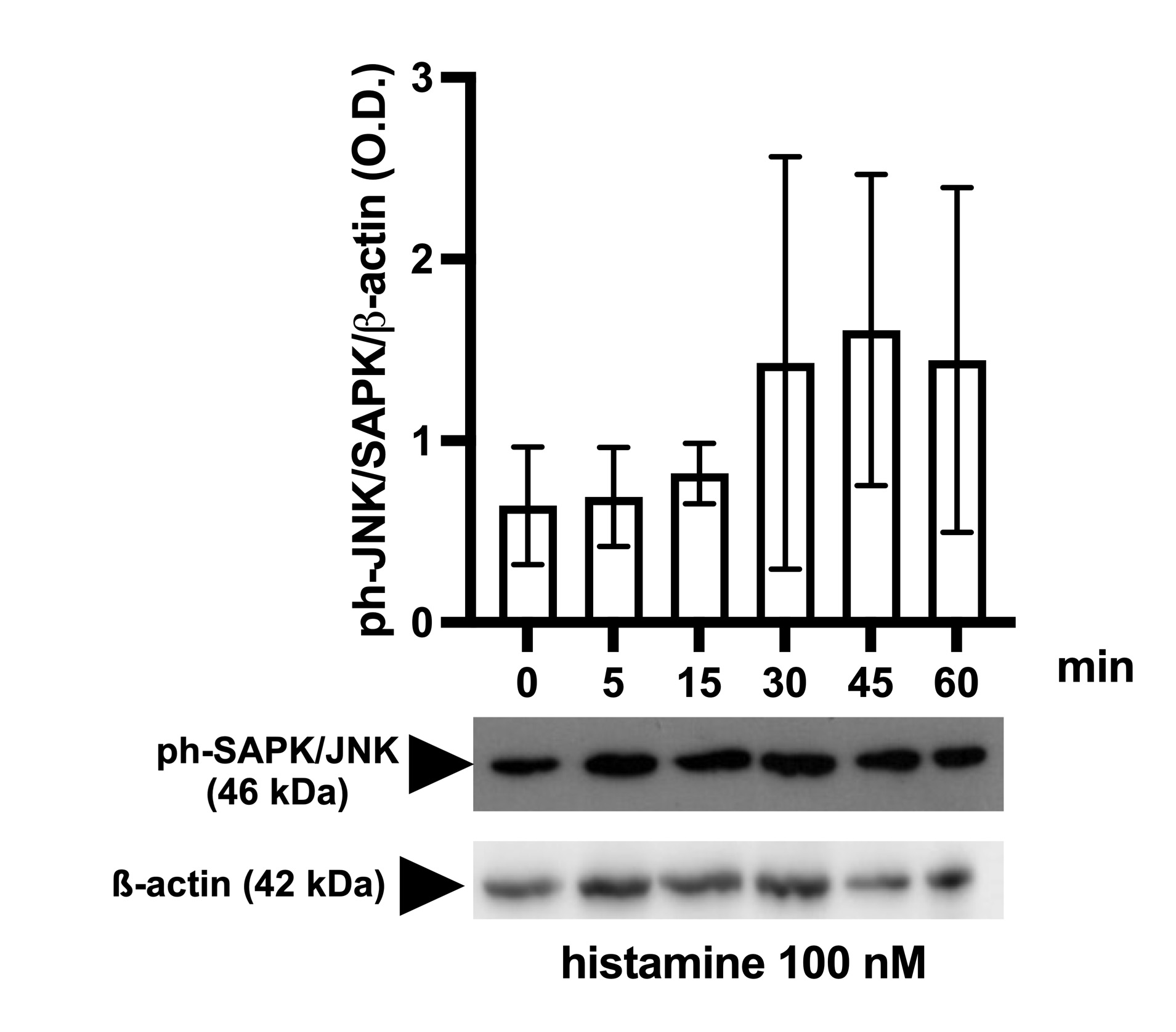

Supplement: Supplementary file 3 — Supplementary Material 3 [file 11_2025_2095_MOESM3_ESM.jpg]
