# Supplementary material for: Histamine regulates the activity and the expression of the Na+/H+ exchanger (NHE)3 in human epithelial HK-2 cells
Source: Inflamm Res. 2025 Sep 12;74(1):122. doi: 10.1007/s00011-025-02095-4 (PMC12432042; doi:10.1007/s00011-025-02095-4)
Supplement: Supplementary file 4 — Supplementary Material 4 [file 11_2025_2095_MOESM4_ESM.docx]

| **Table S_1 Composition in mM of 1X main buffers for the NHE3 activity** | | | | |
| --- | --- | --- | --- | --- |
|  | **HBSS supplementend with HEPES°** | **HCO_3_^-^-free HBSS supplementend with HEPES°** | **NH_4_Cl buffer°** | **Calibration solution*** |
| CaCl_2_ | 1 | 1 | 2 | 1.2 |
| KCl | 5 | 5 | 5 | 135 |
| KH_2_PO_4_ | 0.44 | 0.44 | 1.2 | 2 |
| MgSO_4_ | 0.41 | 0.41 | 1 | 0.8 |
| NaCl | 140 | 140 | 90 | - |
| Na_2_HPO_4_ | 0.34 | 0.34 | - | - |
| NaHCO_3_ | 4.1 | - | - | - |
| D-Glucose | 5.5 | 5.5 | 5.5 | - |
| HEPES | 20 | 20 | 20 | 20 |
| NH_4_Cl | - | - | 20 | - |
| °pH 7.4  *pH adjusted to 6.2, 6.4, 6.6, 6.8, 7.0, 7.2, 7.4, 7.6 by adding HCl or KOH, 10 µM nigericin have been added to each standard  HBSS = Hank's Balanced Salt Solution | | | | |
