# Supplementary material for: Histamine regulates the activity and the expression of the Na+/H+ exchanger (NHE)3 in human epithelial HK-2 cells
Source: Inflamm Res. 2025 Sep 12;74(1):122. doi: 10.1007/s00011-025-02095-4 (PMC12432042; doi:10.1007/s00011-025-02095-4)
Supplement: Supplementary file 5 — Supplementary Material 5 [file 11_2025_2095_MOESM5_ESM.docx]

**Supplementary Material _ Histamine regulates the activity and the expression of the Na^+^/H^+^ exchanger (NHE)3 in human epithelial HK-2 cells**

1. **Supplementary Methods**

***1.1 NHE3 antibody validation by immunoblotting***

HK-2 cells at 80% of confluence were subjected to protein extraction by ice-cold buffer (Tris/HCl 10 mM pH 7.4, NaCl 10 mM, MgCl_2_ 1.5 mM, and 1% Triton X-100), with the addition of Sigmafast, protease inhibitor mixture (PIC 1:1000), phenylmethylsulphonyl fluoride (PMSF, serine protease inhibitor) 1 mM, sodium fluoride (Na_2_F) 1 mM, and sodium orthovanadate 1 mM. Increasing concentrations of proteins (5-40 µg) were subjected to SDS-PAGE using an 8% gel and then transferred to a PVDF membrane at a constant voltage of 100 V for 60 min. The non-specific binding sites were blocked by incubating the PVDF membrane in 5% milk in PBS-Tween 1x for 1 h at room temperature. The membrane was then blotted overnight at 4 °C with either the mouse monoclonal anti-NHE3 antibody (sc-136368) from Santa Cruz Biotechnology or the rabbit polyclonal anti-NHE3 antibody (bs-22546R) from Bioss Inc. β-Actin (1:10000; A2066; Sigma Aldrich) was used as a loading control protein. The respective secondary antibodies (both from Cell Signaling Technology, Inc.) were used at a concentration of 1:3000, and the membranes were incubated for 1 h at room temperature. The membranes were overlaid with Clarity Max Western ECL and visualised using the ChemiDoc™ Touch Imaging System (Bio-Rad).

***1.2 Cell viability assay***

HK-2 cells at 80% of confluence were exposed to vehicle alone, 10 µM of the selective histamine H_1_ receptor antagonist chlorphenamine maleate, or 1 µM of the prototype histamine H_4_ receptor antagonist JNJ7777120 for 48 h. Cell viability was determined by measuring succinate dehydrogenase (SDH) activity using the MTT assay [1].

***1.3 Buffer composition used for NHE3 activity assay***

The compositions of main buffer used to determine the NHE3 activity is reported in Table S_1

| **Table S_1 Composition in mM of 1X main buffers for the NHE3 activity** | | | | |
| --- | --- | --- | --- | --- |
|  | **HBSS supplementend with HEPES°** | **HCO_3_^-^-free HBSS supplementend with HEPES°** | **NH_4_Cl buffer°** | **Calibration solution*** |
| CaCl_2_ | 1 | 1 | 2 | 1.2 |
| KCl | 5 | 5 | 5 | 135 |
| KH_2_PO_4_ | 0.44 | 0.44 | 1.2 | 2 |
| MgSO_4_ | 0.41 | 0.41 | 1 | 0.8 |
| NaCl | 140 | 140 | 90 | - |
| Na_2_HPO_4_ | 0.34 | 0.34 | - | - |
| NaHCO_3_ | 4.1 | - | - | - |
| D-Glucose | 5.5 | 5.5 | 5.5 | - |
| HEPES | 20 | 20 | 20 | 20 |
| NH_4_Cl | - | - | 20 | - |
| °pH 7.4  *pH adjusted to 6.2, 6.4, 6.6, 6.8, 7.0, 7.2, 7.4, 7.6 by adding HCl or KOH, 10 µM nigericin have been added to each standard  HBSS = Hank's Balanced Salt Solution | | | | |

***1.4 Immunoblotting for JNK activation***

Proteins were extracted from HK-2 cells challenged with histamine (100 nM 0-60) min by ice-cold buffer (Tris/HCl 10 mM pH 7.4, NaCl 10 mM, MgCl_2_ 1.5 mM, and 1% Triton X-100), with the addition of Sigmafast, protease inhibitor mixture (PIC 1:1000), phenylmethylsulphonyl fluoride (PMSF, serine protease inhibitor) 1 mM, sodium fluoride (Na_2_F) 1 mM, and sodium orthovanadate 1 mM. Twenty μg of protein was subjected to SDS-PAGE using a 10 % gel and then transferred onto a PVDF membrane at a constant voltage of 100 V for 60 min. After blocking the non-specific binding sites with 5% milk in PBS-Tween 1x for 1 h at room temperature, the PVDF membrane was incubated overnight at 4°C with ph-SAPK/JNK (1:1000; Thr183/Tyr185; 81E11; #4668, Cell Signaling Technology, Inc.). β-Actin (1:10000) was used as a loading uniformity control protein. The respective secondary antibodies were used at a concentration of 1:3000 and incubated for 1 h at room temperature. The membranes were overlaid with WesternBright™ Quantum and then exposed to Hyperfilm ECL. Densitometric analysis was performed on digitised images using the ImageJ software package. The optical density (OD) values were calculated from the background-corrected band intensity and area of the targeted protein. Normalisation was performed by dividing the OD values of the target protein by the OD of the relative β-actin.

1. **Supplementary Results**

***2.1 NHE3 antibody validation by immunoblotting***

The expression of NHE3 in HK-2 cells was confirmed by immunoblotting. This was achieved by comparing the results obtained using two different antibodies against the human NHE3 protein: the mouse monoclonal anti-NHE3 antibody (sc-136368) from Santa Cruz Biotechnology and the rabbit polyclonal anti-NHE3 antibody (bs-22546R) from Bioss Inc. As shown in Fig. S_1, immunoblotting with increasing concentrations of protein extract (5–40 µg) revealed a 100-kDa band following incubation with both antibodies. This corresponded to the predicted molecular weight of NHE3 (93, 80-100 kDa). The rabbit polyclonal anti-NHE3 antibody (bs-22546R) from Bioss Inc. revealed a multiband spectrum, with a high signal also present at 130 kDa.


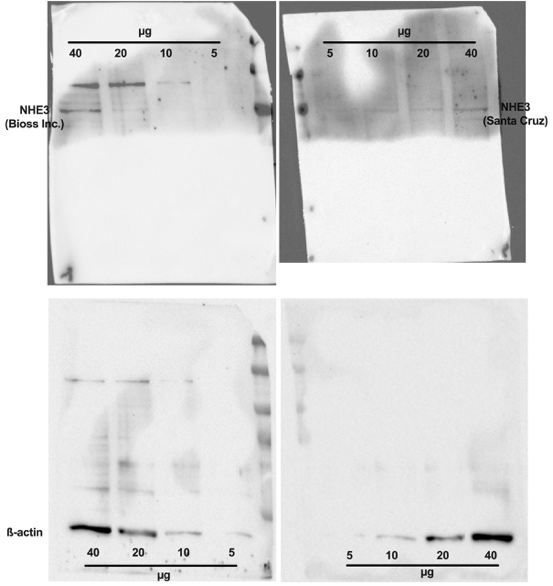


**Fig S_1** **NHE3 expression in HK-2 cells: antibody validation.** Representative composite image (colourimetric and chemiluminescence) of immunoblotting to detect NHE3 (93, 80- 100 kDa) using either the rabbit polyclonal anti-NHE3 antibody (bs-22546R) from Bioss Inc., or the mouse monoclonal anti-NHE3 antibody (sc-136368) from Santa Cruz Biotechnology. β-Actin was used as a loading control protein.

***2.2 Effect of chlorphenamine maleate and JNJ7777120 on cell viability***

To confirm HK-2 cell viability following exposure to 10 µM chlorphenamine maleate and 1 µM JNJ7777120, the respective drugs were administered to the cells for 48 hours. As illustrated in Fig. S_2, neither drug affected cell viability as determined by the MTT assay.


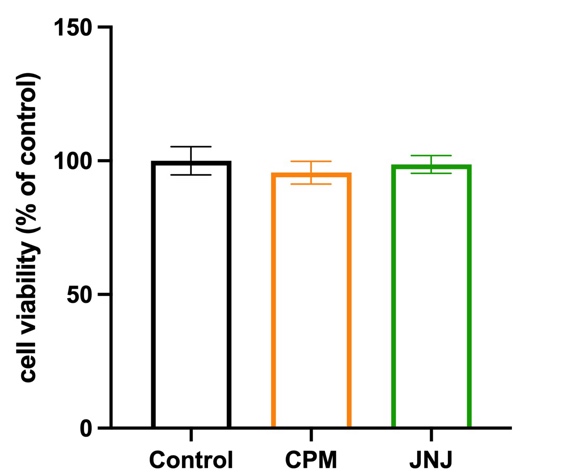


**Fig S_2 Effect of chlorphenamine maleate and JNJ7777120 on cell viability.** The histograms show the percentage of viable cells compared to the control condition after HK-2 cells were exposed to 10 µM of chlorphenamine maleate (CPM) or 1 µM of JNJ7777120 (JNJ) for 48 hours. Cell viability was measured using the MTT assay at the end of the experiment.

***2.3 JNK/SAPK involvement in NHE3 expression and activation induced by histamine***

HK2 cells were exposed to 100 nM additional histamine for 0–60 minutes. As Fig. S_3 shows, exposure to histamine at different time points does not induce JNK/SAPK phosphorylation, suggesting that only p38 MAPK and ERK1/2 are involved in histamine-induced NHE3 activation (see Fig. 8 in the manuscript).

**
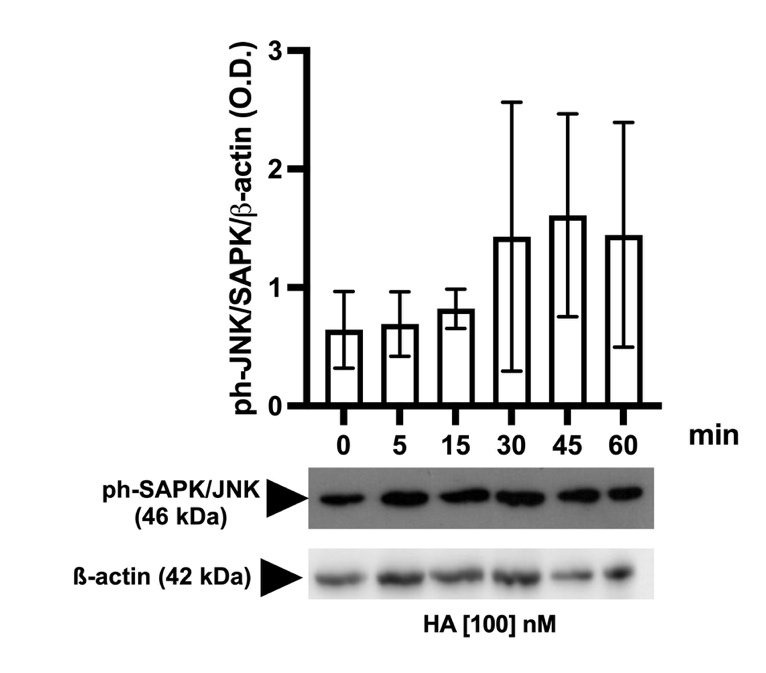
**

**Fig S_3 JNK/SAPK involvement in the NHE3 regulation elicited by histamine.** Densitometric analysis of the effect of additional histamine (HA, 100nM) on the phosphorylation of SAPK/JNK over 0-60 minutes. The results, expressed as optical density (O.D.), are the mean ± SEM of three independent experiments normalised for β-actin. * *P* ≤ 0.05, *** *P* ≤ 0.001. The radiograph image shows the immunoblotting bands corresponding to the expression of ph-SAPK/JNK and the β-actin control protein.

**Supplementary References**

1. Mosmann T. Rapid colorimetric assay for cellular growth and survival: application to proliferation and cytotoxicity assays. J Immunol Methods 1983; 65:55-63.
