# Supplementary material for: Histamine regulates the activity and the expression of the Na+/H+ exchanger (NHE)3 in human epithelial HK-2 cells
Source: Inflamm Res. 2025 Sep 12;74(1):122. doi: 10.1007/s00011-025-02095-4 (PMC12432042; doi:10.1007/s00011-025-02095-4)
Supplement: Supplementary file 6 — Supplementary Material 6 [file 11_2025_2095_MOESM6_ESM.pdf]

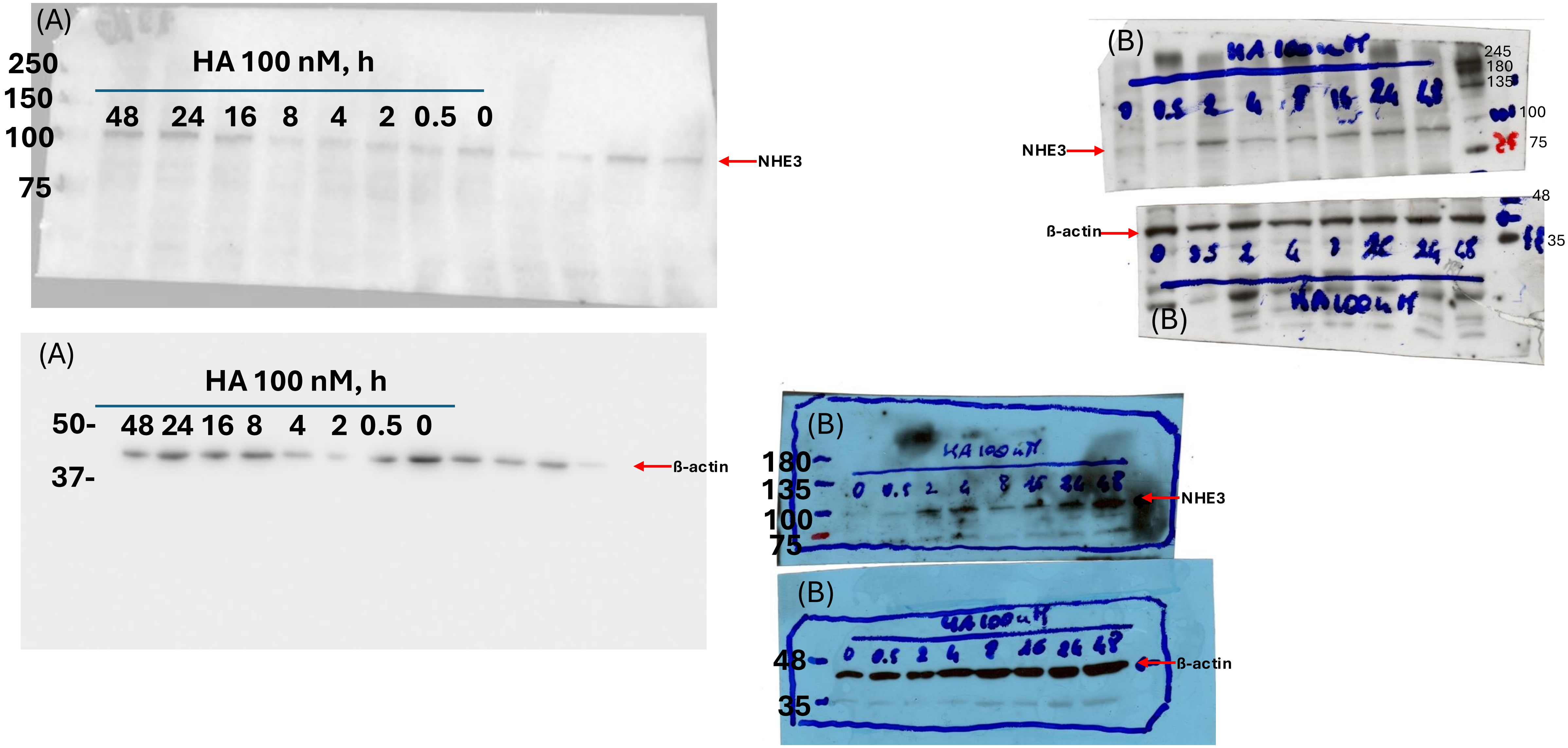

Fig S2_1 Original western blots from 3 independent experiments for histamine effect on NHE3 protein expression shown in Fig 4A. NHE3 protein expression in HK-2 cells exposed to additional 100 nM histamine (HA) for 0-48 h. Proteins were resolved in a 8 % SDS-PAGE gel. Molecular weight standards: prestained Precision Plus Protein™ WesternC™ Protein Standards (10 - 250 kDa - 1610399, Bio-Rad Laboratories, Inc.) or Opti-Protein XL Marker (10 - 245 kDa - G266, Applied Biological Materials Inc.). (A) Composite images captured with CCD camera. (B) X-ray film developed in dark room.

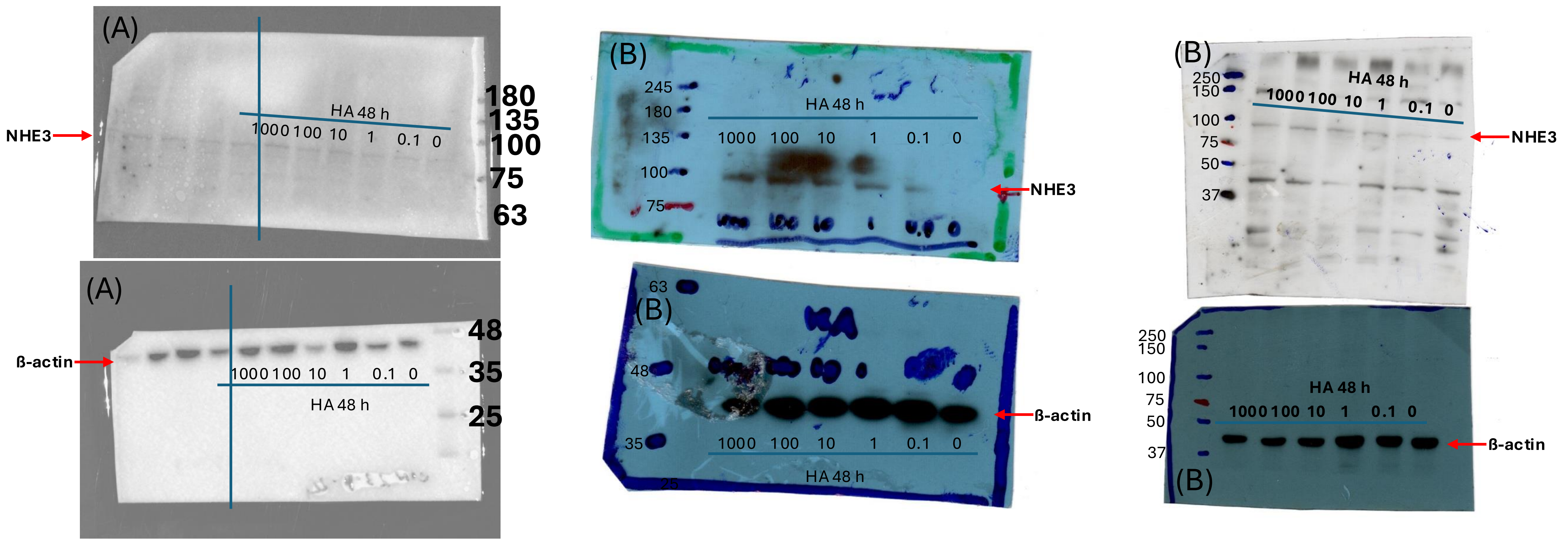

Fig S2_2 Original western blots from 3 independent experiments for histamine effect on NHE3 protein expression shown in Fig 4BNHE3 expression in HK-2 cells exposed to additional 0-1000 histamine (HA) nM for 48 h. Proteins were resolved in a 8 % SDS-PAGE gel. Molecular weight standards: Precision Plus Protein™ WesternC™ Protein Standards (10 - 250 kDa - 1610399, Bio-Rad Laboratories, Inc.) or Opti-Protein XL Marker (10 - 245 kDa - G266, Applied Biological Materials Inc.) (A) Composite images captured with CCD camera. (B) X-ray film developed in dark room.

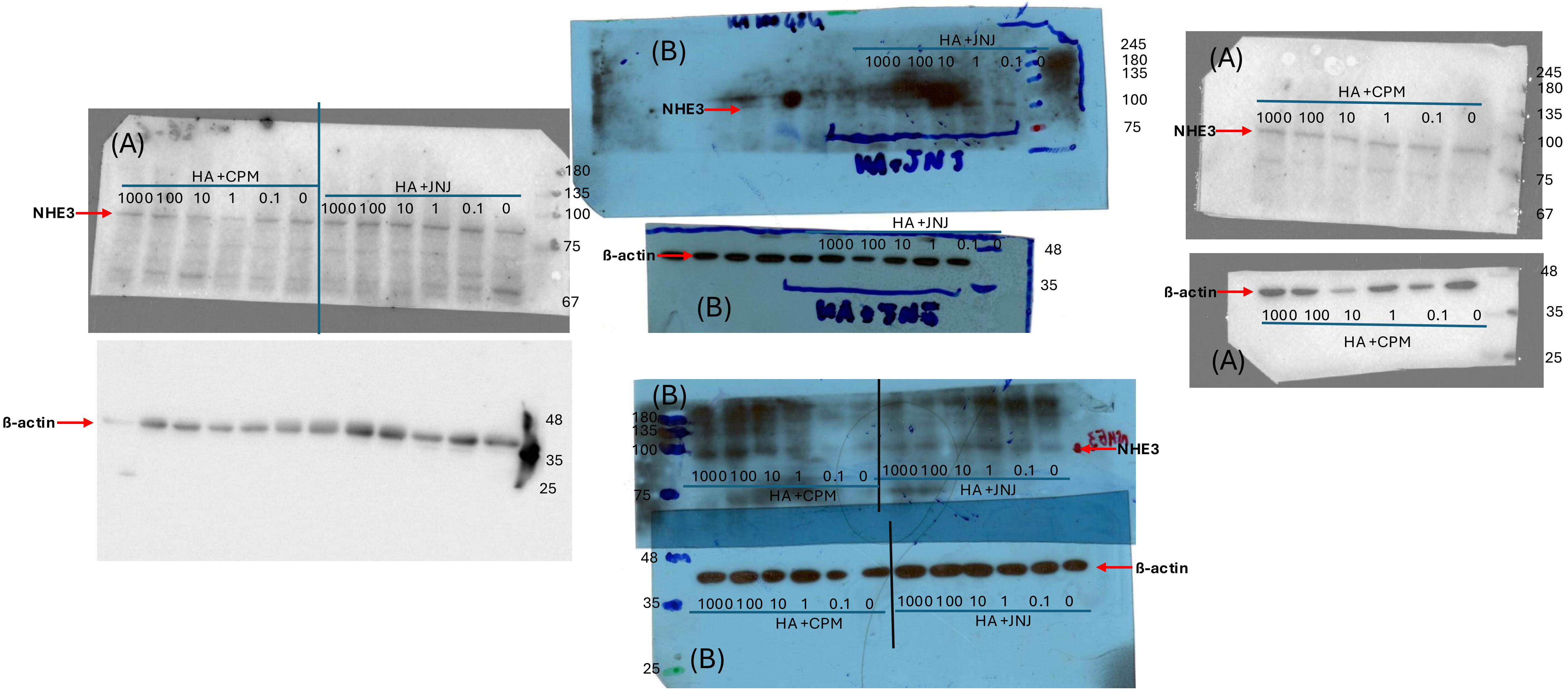

Fig S2_3 Original western blots from 3 independent experiments for chlorphenamine 10 µM and JNJ-7777120 1 µM effect on NHE3 expression shown in Fig 7A. NHE3 expression in HK-2 cells exposed to 0-1000 histamine (HA) nM for 48h in the presence of chlorphenamine (CPM) 10 µM or JNJ-7777120 (JNJ) 1 µM. Original data for histamine alone are shown in Fig S2_2. Proteins were resolved in a 8 % SDS-PAGE gel. Molecular weight standards: Opti-Protein XL Marker (10 - 245 kDa - G266, Applied Biological Materials Inc.). (A) Composite images captured with CCD camera. (B) X-ray film developed in dark room.

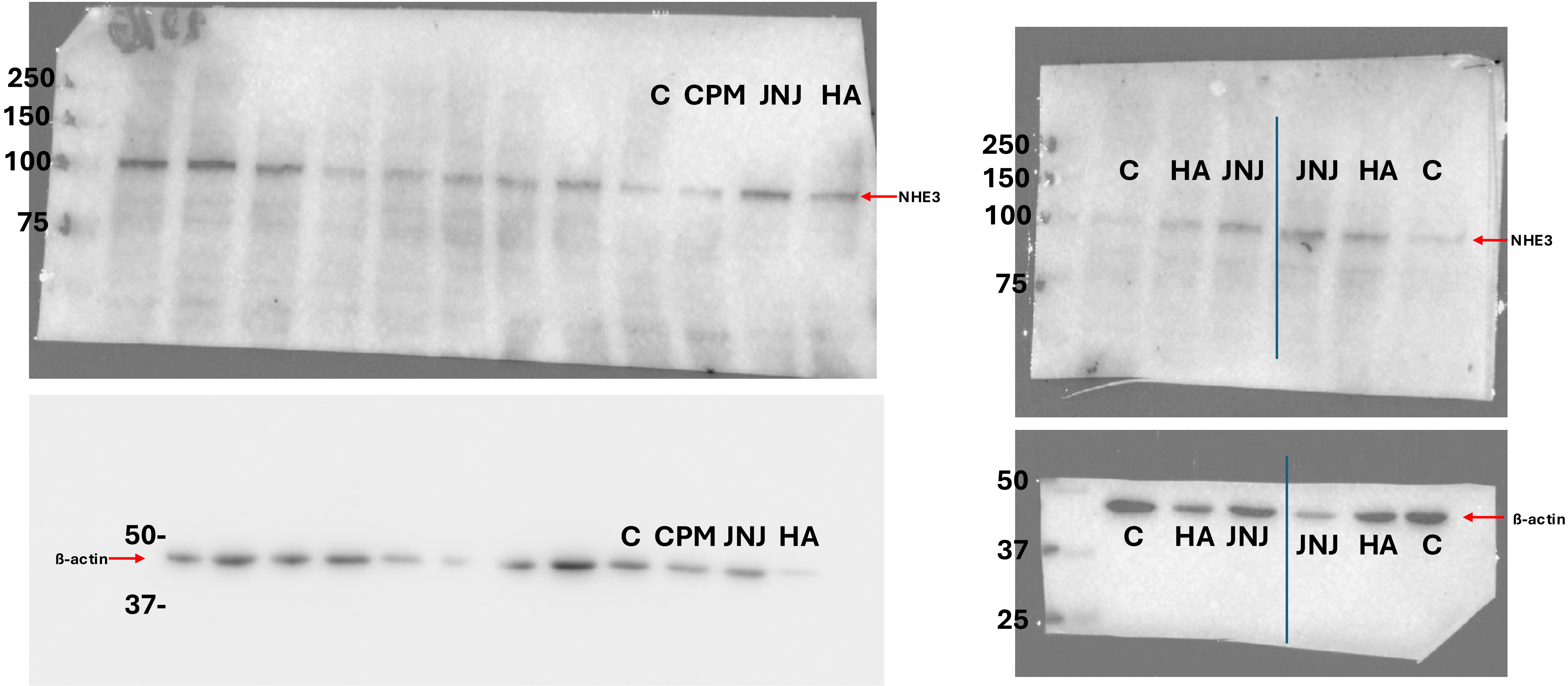

Fig S2_4 Original western blots from 3 independent experiments for chlorphenamine 10 µM and JNJ-7777120 1 µM effect on NHE3 expression shown in Fig 7B. Composite images captured with CCD camera of NHE3 expression induced by additional 100 nM histamine (HA) or JNJ-7777120 (JNJ) 1µM for 48 h. Proteins were resolved in a 8% SDS-PAGE gel. Molecular weight standards: prestained Precision Plus Protein™ WesternC™ Protein Standards (10 - 250 kDa - 1610399, Bio-Rad Laboratories, Inc.)

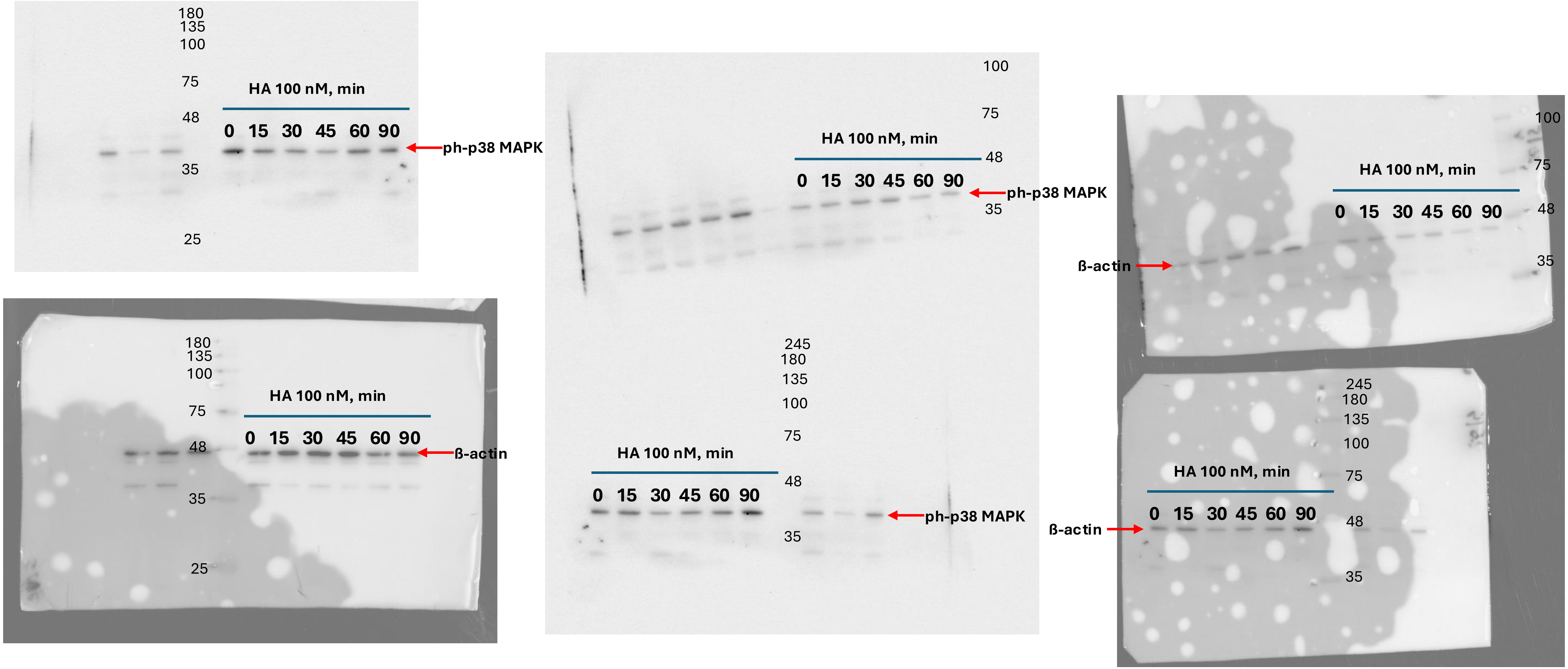

Fig S2_5 Original western blots from 3 independent experiments for MAPK involvement in the NHE3 regulation elicited by histamine shown in Fig 9A. Composite images captured with CCD camera to evaluate the effect evoked by additional 100 nM histamine for 0 - 90 min on the phosphorylation of p38 MAPK. Proteins were resolved in a 10% SDS-PAGE gel. Molecular weight standards: Opti-Protein XL Marker (10 - 245 kDa - G266, Applied Biological Materials Inc.)

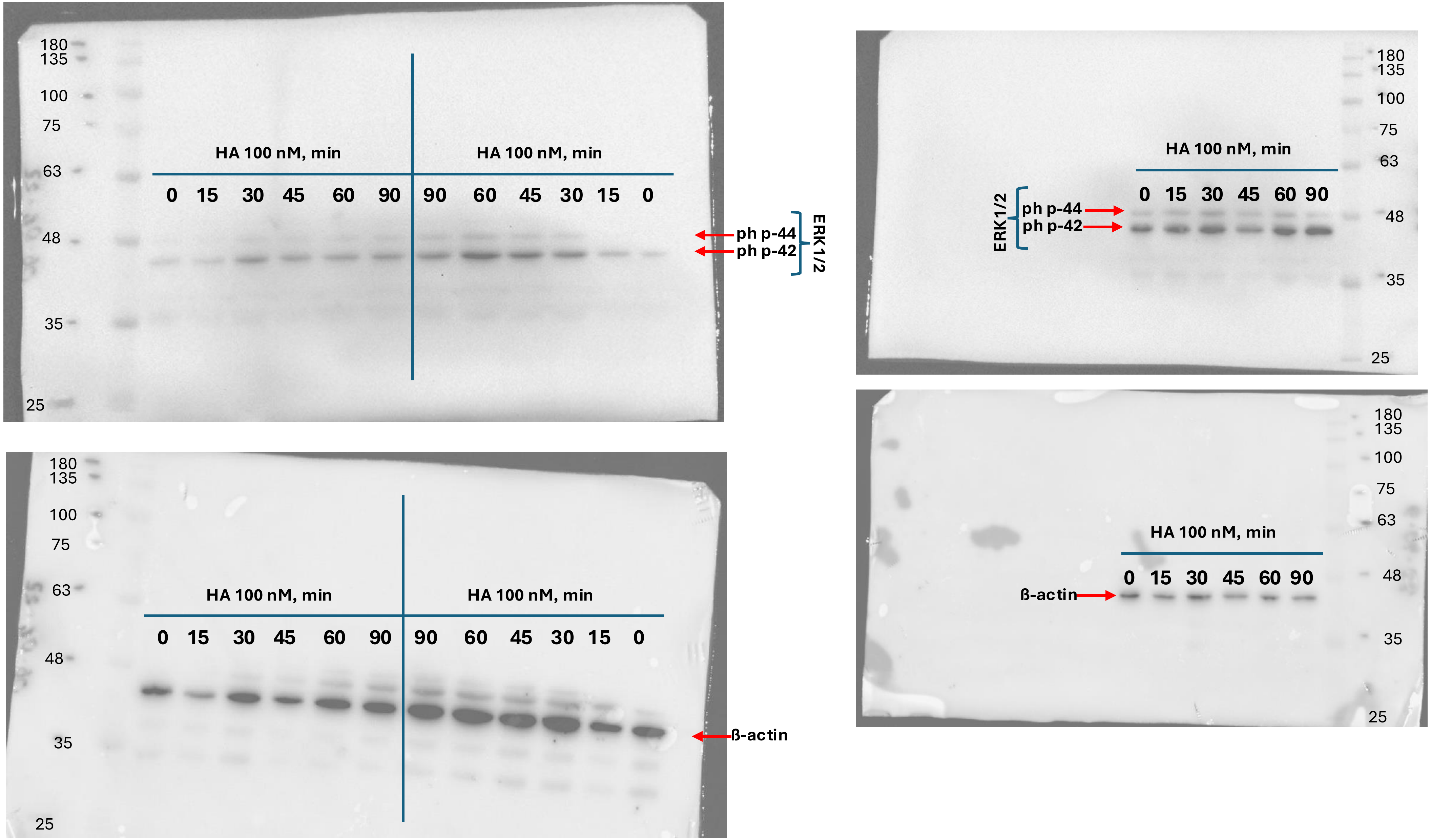

Fig S2_6 Original western blots from 3 independent experiments for ERK1/2 involvement in the NHE3 regulation elicited by histamine shown in Fig 9A. Composite images captured with CCD camera to evaluate the effect evoked by additional 100 nM histamine for 0 - 90 min on the phosphorylation of ERK1/2. Proteins were resolved in a 10% SDS-PAGE gel. Molecular weight standards: Opti-Protein XL Marker (10 - 245 kDa - G266, Applied Biological Materials Inc.)

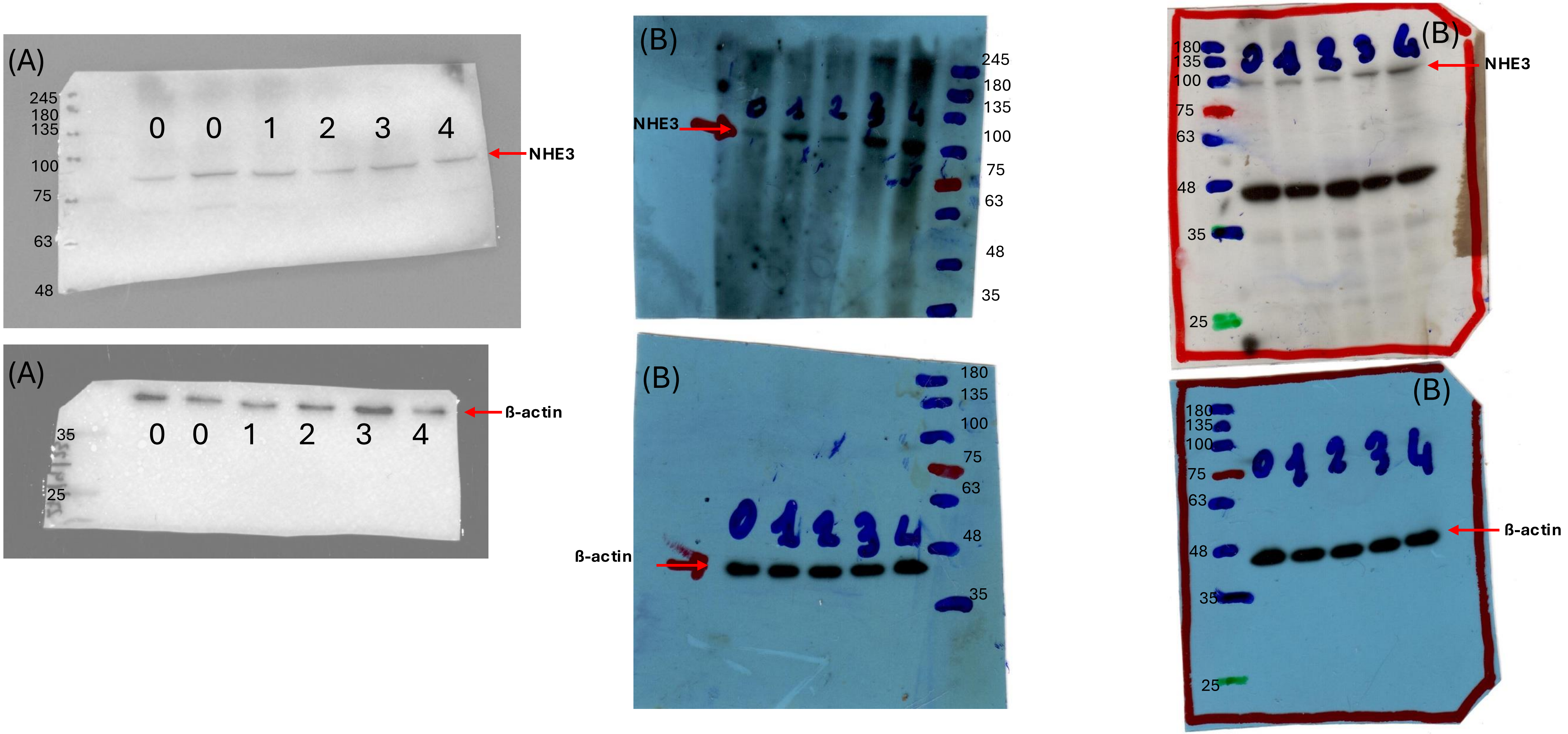

Fig S2_7 Original western blots from 3 independent experiments for MAPK involvement in the NHE3 regulation elicited by histamine shown in Fig 9C. NHE3 expression after MAPK inhibition by SB202190 (p38 MAPK), PD98059 (ERK1/2), or SP600125 (SAPK/JNK) 20 µM for 30 min before challenging with histamine (HA) 100 nM for 48 h. Samples rapresents: 0 = control, 1 = HA 100 nM 48 h, 2 = SB20219 20 µM for 30 min + HA 100 nM 48 h, 3 = PD98059 20 µM for 30 min + HA 100 nM 48 h, 4= SP600125 20 µM for 30 min + HA 100 nM 48 h. Proteins were resolved in a 8 % SDS-PAGE gel. Molecular weight standards: Opti-Protein XL Marker (10 - 245 kDa - G266, Applied Biological Materials Inc.). (A) Composite images captured with CCD camera. (B) X-ray film developed in dark room.

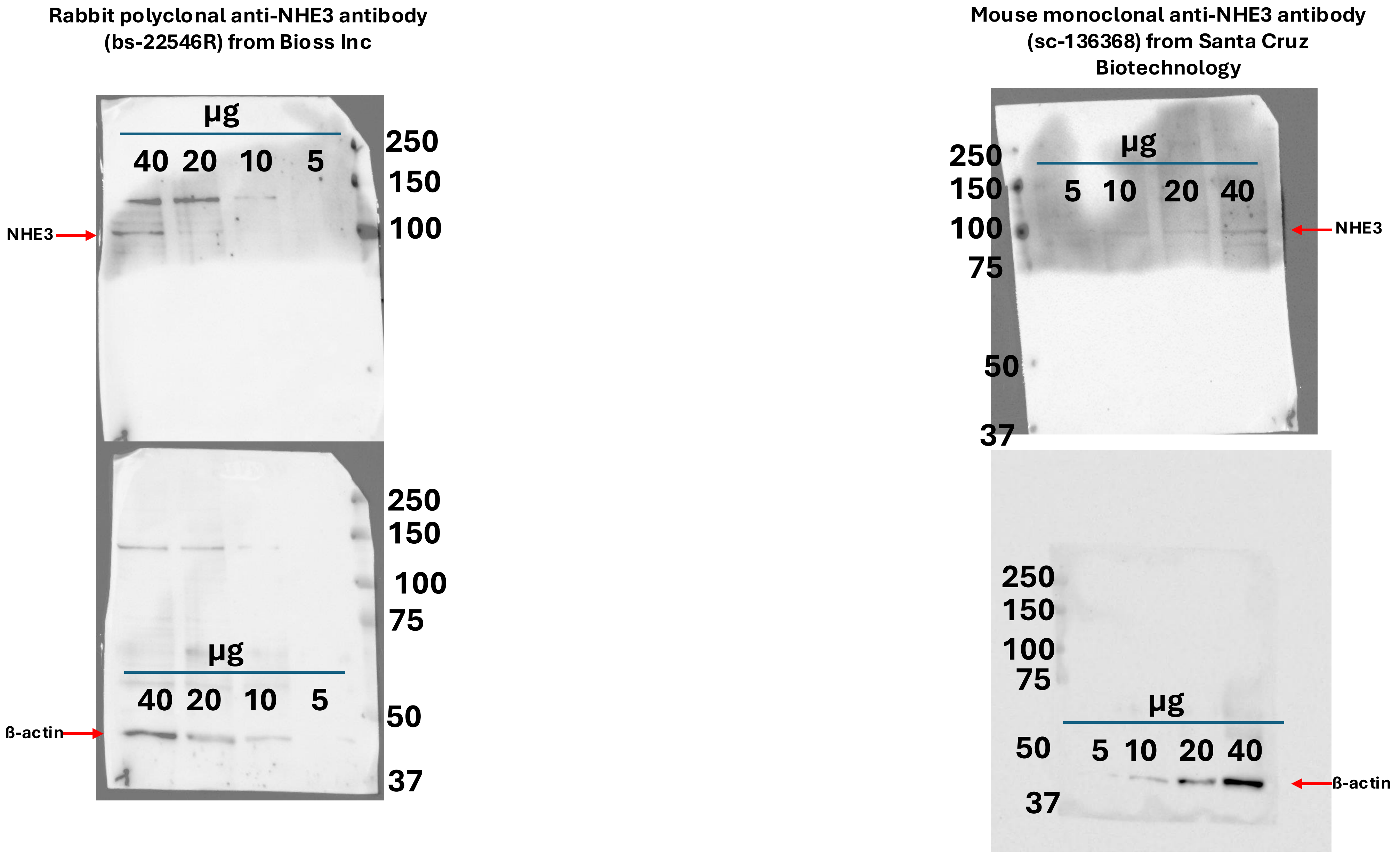

Fig S2_8 Original western blots for antibody validation shown in Fig S_1. NHE3 (93, 80- 100 kDa) detection using increasing concentrations of protein extract (5–40 µg) rom HK-2 cellss using either the rabbit polyclonal anti-NHE3 antibody (bs-22546R) from Bioss Inc., or the mouse monoclonal anti-NHE3 antibody (sc-136368) from Santa Cruz Biotechnology. Proteins were resolved in a 8 % SDS-PAGE gel. Molecular weight standards: prestained Precision Plus Protein™ WesternC™ Protein Standards (10 - 250 kDa - 1610399, Bio-Rad Laboratories, Inc.)

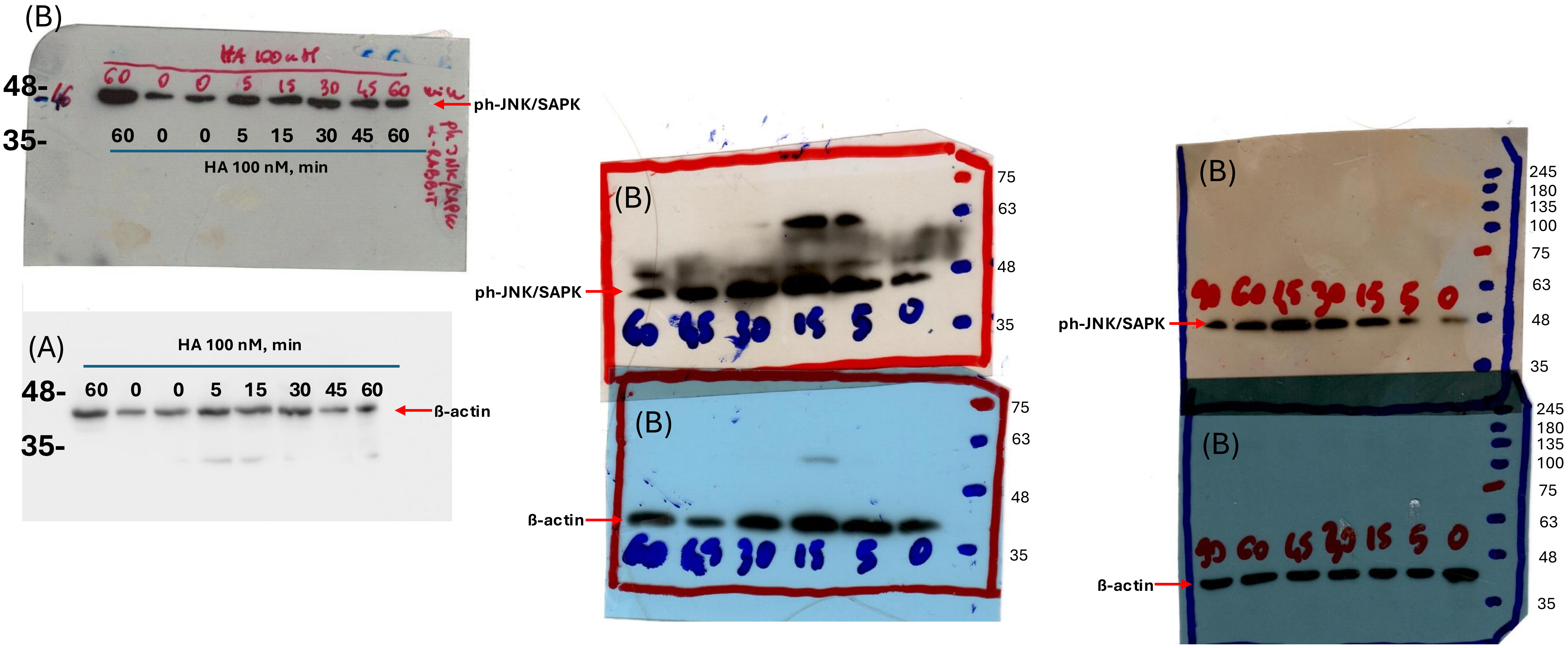

Fig S2_9 Original western blots from 3 independent experiments for JNK/SAPK involvement in the NHE3 regulation elicited by histamine in Fig S_3. Composite images captured with CCD camera (A) or X-ray films (B) showing the effect of additional 100 nM histamine (HA) on the phosphorylation of SAPK/JNK over 0-60. Proteins were resolved in a 10% SDS-PAGE gel. Molecular weight standards: Opti-Protein XL Marker (10 - 245 kDa - G266, Applied Biological Materials Inc.)
